# Arthropod abundances track soil fertility across a lowland tropical forest landscape

**DOI:** 10.1111/1365-2656.70060

**Published:** 2025-05-23

**Authors:** Orpheus M. Butler, Vanessa Sanchez, Kara J. Simpson, Donald Windsor

**Affiliations:** ^1^ Smithsonian Tropical Research Institute Panama Panama; ^2^ The University of Panama Panama Panama; ^3^ Present address: Australian Rivers Institute and School of Environment and Science Griffith University Brisbane Queensland Australia

**Keywords:** Barro Colorado Natural Monument, Coleoptera, geodiversity, geoecology, Gigante Peninsula, phosphorus

## Abstract

1. Soil phosphorus (P) drives productivity and floristic composition across tropical forest landscapes, but equivalent links between soil P and tropical forest fauna remain poorly understood.
2. We evaluated soil P as a driver of understorey Coleoptera and epigeal arthropod assemblages across a natural landscape‐level soil fertility gradient and at an adjacent site‐level P fertilisation experiment in central Panama.
3. A fifth of Coleoptera families in flight‐intercept traps (corresponding to 10%–55% [range of values across 10 sites] of all specimens), a third of litter‐extracted Coleoptera families (7%–86% of specimens), and almost half of litter‐extracted fauna orders (20%–69% of specimens) displayed significant abundance trends across the natural fertility gradient. These responses were not paralleled in the site‐level fertilisation experiment, which could be an indication that floristic composition is a proximal driver of arthropod–soil P associations across the lowland tropical forest landscape of central Panama.
4. By revealing the significant, indirect role of soil P in shaping tropical arthropod assemblages, our results highlight the ongoing connection between geological‐scale processes and the contemporary ecology of the most diverse group of animals on Earth.

## Graphical abstract

This study reveals strong, diverse associations between soil phosphorus and the abundances of understorey arthropods across a lowland tropical forest landscape. These patterns were not paralleled in an adjacent site‐level fertilisation experiment, raising the interesting possibility that arthropod–phosphorus associations could be mediated by spatial turnover of tropical tree species.

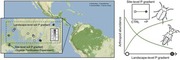

## 1 INTRODUCTION

The soils of lowland tropical forests are subject to intense weathering due to the warm and wet climatic conditions that prevail at Earth's low latitudes. As a result, most lowland tropical forest soils contain low levels of phosphorus (P) (Cunha et al., 2022; Turner & Engelbrecht, 2011)—a mineral‐derived nutrient that comprises a key constituent of various biomolecules (e.g. nucleic acids and membrane phospholipids) and is, therefore, essential to all lifeforms (Sterner & Elser, 2002). Prior studies indicate that soil P influences primary productivity and floristic composition in tropical forests as well as the physiology of individual tropical plant species (Condit et al., 2013; Santiago et al., 2012; Turner et al., 2018). Plants, in turn, constitute the base of tropical forest food webs, providing the ultimate source of carbon (C) and soil‐derived nutrients (including P) to herbivorous and detritivorous organisms and higher order consumers (Kaspari & Yanoviak, 2009). However, the low‐P status of most tropical soils means that first‐order consumers in the tropics often face a large stoichiometric mismatch for P, with the extent of this mismatch determined partly by soil P availability and the effects thereof on the stoichiometric quality and quantity of plant‐based resources. It is, therefore, logical to expect assemblages of arthropods, micro‐organisms and other heterotrophic taxa in lowland tropical forests to be influenced by soil P, with this influence potentially mediated by vegetation. However, few studies have examined the potential links between soil P and the assemblages of heterotrophic organisms in tropical forests, and the consequent knowledge gap is particularly large in the case of arthropods—the most species‐rich animal taxon on Earth.

Evidence from the handful of prior studies indicates that arthropod abundance, biomass and diversity are often positively associated with soil and/or litter P content in tropical forests (Bujan et al., 2016; Jochum et al., 2017; Kaspari et al., 2017; McGlynn et al., 2007; Tarli et al., 2014), with negative responses comparatively uncommon (but see Bujan et al., 2016; McGlynn et al., 2009). Several hypotheses have been invoked to explain the positive arthropod–soil P associations observed in these studies (Bujan et al., 2016; Kaspari & Yanoviak, 2009; Sayer et al., 2010), and each of these is ultimately predicated upon a state of P limitation (either direct; e.g. the ‘structural shortage hypothesis’; Kaspari & Yanoviak, 2009) or indirect (i.e. the ‘secondary productivity’ and ‘ecosystem size’ hypotheses; e.g. Kaspari & Yanoviak, 2009) of some or all constituents of the arthropod assemblages in question, with corresponding consequences for abundance/biomass, diversity and community composition emerging from inter‐taxon variation in the degree of said P limitation (e.g. Bujan et al., 2016; Butler et al., 2021). Under the structural shortage hypothesis, P directly constrains the individual and/or population growth of a given taxon because environmental P availability is insufficient to meet the demands of the taxon's physiology. Under the secondary productivity hypothesis, the response variable is controlled by availability of food resources, such as microbial biomass, which itself is explicitly P‐limited, while under the ecosystem size hypothesis, P constrains rates of primary productivity and decomposition, thereby controlling the absolute habitat volume and the abundance and diversity of fauna. However, when carried out over large spatial areas or wide soil/resource P gradients, tests of these hypotheses are potentially complicated by confounding factors, such as the correlation of P with various other soil properties and the strong spatial turnover of tropical tree species across gradients of soil fertility (Condit et al., 2013; Turner et al., 2018). One way to partially resolve such confoundment is to compare natural landscape‐level gradients to experimental soil P gradients within the same landscape. With this approach, landscape‐level gradients reveal patterns present in nature, whereas site‐level experimental gradients isolate soil P availability from other potentially confounding variables, including covarying soil properties and floristic composition (which is not immediately altered by soil P fertilisation in the case of tropical forests (Wright et al., 2018)).

**FIGURE 1:**
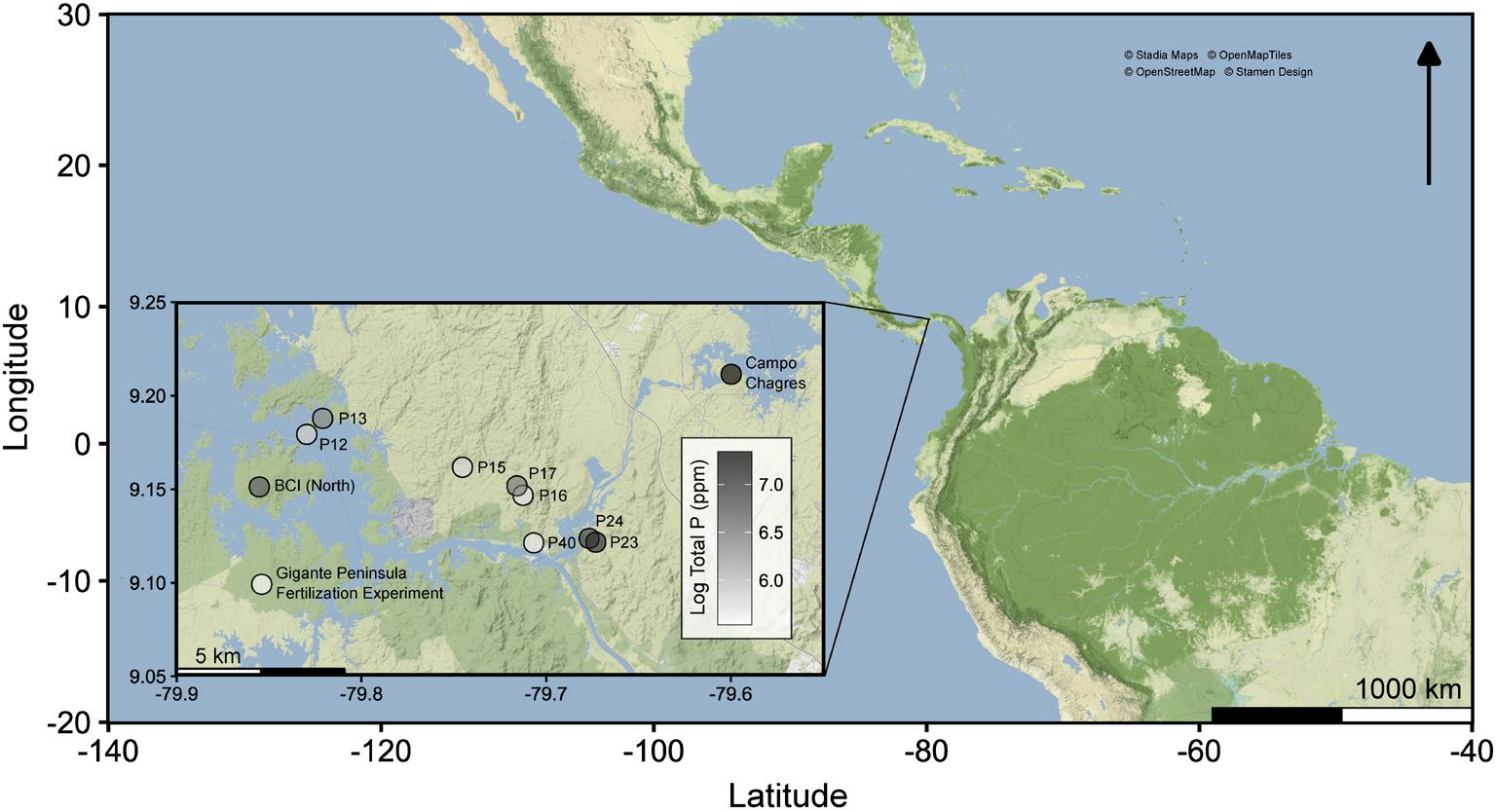
Map of study plots in central Panama. Symbol fills indicate soil (0–10 cm) total P content (mg P kg soil^−1^; range of untransformed values: 255–1542 mg P kg soil^−1^).

Thus, to evaluate the role of soil P as a driver of arthropod abundance, diversity and assemblage composition, along with the potential mechanisms contributing to this role, we carried out surveys of understorey arthropod fauna, with a focus on Coleoptera, across a natural landscape‐level soil P gradient in the lowland tropical forests of central Panama and at an adjacent site‐level soil P fertilisation experiment on Gigante Peninsula (Figure 1). We did not directly test the effect of floristic turnover as a driver of arthropod assemblages; however, the parallel surveys of natural and experimental soil P fertility gradients provide an opportunity to isolate the role of P and clarify, to an extent, the mechanism by which P exerts its influence.

The natural soil fertility gradient in central Panama is underpinned by the dynamic geological history of the Panamanian Isthmus, which has given rise to a variety of soil types within a relatively small area (Stewart et al., 1980; Turner & Engelbrecht, 2011). The P content of plant and microbial biomass is positively associated with soil P across this gradient (Dietterich et al., 2022; Santiago et al., 2005; Townsend et al., 2007), as is primary productivity (Turner et al., 2018), and these effects are coupled to marked shifts in floristic composition (Condit et al., 2013; Turner et al., 2018; Umaña et al., 2021). Meanwhile, the long‐running forest fertilisation experiment at Gigante Peninsula provides a controlled, site‐level test of arthropod assemblage responses to soil P specifically. According to measurements made 12–17 years prior to the commencement of our study (after 4–9 years of annual P addition), the P fertilisation treatment has increased surface soil total P content ~1.4‐fold, Mehlich‐extractable PO_4_^3−^–P ~29‐fold, and resin‐extractable PO_4_^3−^–P 45‐fold (Turner et al., 2013; Yavitt et al., 2011). Likewise, P fertilisation has increased the P contents of plant material and soil microbial biomass (Kaspari et al., 2008; Santiago et al., 2012; Schreeg et al., 2014; Turner & Wright, 2014; Wright et al., 2024; Wurzburger & Wright, 2015), the rates of fine litter production (Wright et al., 2011) and species‐specific flower and fruit production (Fortier & Wright, 2021; Kaspari et al., 2008), and the total quantity of microbial biomass in soil (Turner & Wright, 2014). Thus, both the quality and quantity of several major arthropod food resource classes have been increased by the P addition treatment at Gigante.

We hypothesised that understorey arthropods in lowland tropical forest are broadly P‐limited, either directly or indirectly, such that the size of arthropod assemblages should vary across landscape‐level gradients of soil fertility, increasing from low‐fertility sites to high‐fertility sites, with corresponding covariation in the diversity of arthropod assemblages. We further hypothesised that the various constituent taxa or functional groups comprising arthropod assemblages vary in the strength of their P limitation, leading to significant variation in the strength of taxon‐specific abundance–soil P associations on the landscape level, and significant, corresponding shifts in community composition from low‐fertility to high‐fertility sites. Finally, we hypothesised that at least some of the variation in arthropod abundance, diversity and composition across natural landscape‐level gradients of soil fertility is due to parallel variation in floristic composition; thus, responses of arthropod assemblages to natural soil fertility gradients should be stronger than responses to experimental soil fertilisation at the site level.

## 2 MATERIALS AND METHODS

### 2.1 Study design and site descriptions

Extensive soil data are available for numerous established research plots in central Panama, including data for multiple indices of soil P (resin‐extractable PO_4_^3−^, Mehlich‐3 extractable PO_4_^3−^ and total P; Figure S1; see Condit et al., 2013 for details of soil chemical analyses). We selected 10 plots that spanned a 5.5‐fold gradient of soil total P and an 85‐fold gradient of resin P (Figure 1; Table 1). Low‐P sites were partially interspersed with high‐P sites, and Mantel tests found no evidence for spatial autocorrelation of soil P (Mantel *p* values >0.05), such that spatial proximity of plots is unlikely to confound any influence of soil P. We aimed to eliminate potential confoundment by rainfall and elevation. Thus, for the plots we selected, modelled values of annual precipitation were not correlated with soil P (*R* ^2^ < 0.1) and varied just 1.2‐fold, ranging 2154–2595 mm across our plot selection (Figure S2). Elevation was likewise orthogonal to soil P (*R* ^2^ < 0.1; Figure S2). We also tried to minimise collinearity between soil P and other nutrients through careful plot selection based on prior knowledge of soil properties. For total P, the most strongly collinear nutrient across our plots was Ca (*R* ^2^ = 0.63) followed by total N (*R* ^2^ = 0.55) and KCl‐extractable NO_3_^−^ (*R* ^2^ = 0.43); other nutrients (Al, K, Fe, Mg, Mn, Zn, NH_4_^+^) were not correlated with soil total P (*R* ^2^ < 0.2).

**TABLE 1:** Soil information for 10 lowland tropical forest plots in central Panama that comprise a strong natural soil phosphorus fertility gradient^a^ and for the control treatment of the Gigante Peninsula^b^.

Site	Geological parent material	Soil order	Total P (mg P kg soil^−1^)	Mehlich P (mg P kg soil^−1^)	Resin P (mg P kg soil^−1^)
Cerro Pelado	Bas Obispo formation, Oligocene agglomerate and hard tuff	Undescribed	282	0.76	0.27
P16	Pre‐tertiary basalt	Inceptisols	306	1.87	0.66
P15	Gatuncillo formation; mudstone, siltstone, quartz, sandstone, algal and foraminiferal limestone	Alfisols	319	1.56	1.20
P12	Bohio formation; volcanic conglomerate, principally basaltic and graywacke sandstone	Inceptisols	339	2.54	1.60
P13	Caimito formation; tuffaceous sandstone, tuffaceous siltstone, tuff and foraminiferal limestone	Inceptisols	552	4.33	8.44
P17	Altered basaltic and andesitic lavas and tuff, including dioritic and dacitic intrusive rocks	Inceptisols	614	2.95	4.81
BCI	Andesitic flow; tuffaceous sandstone, tuffaceous siltstone, tuff and foraminiferal limestone	Oxisols	771	3.12	1.86
P24	Las Cascadas formation; agglomerate of tuffaceous siltstone, tuff and foraminiferal limestone	Alfisols	940	5.29	13.14
P23	Igneous sedimentary; agglomerate of tuffaceous siltstone, tuff and foraminiferal limestone	Alfisols	1160	21.97	22.36
Campo Chagres	Upper Alhajuela formation; calcareous sandstone, tuffaceous sandstone and limestone, late Early Miocene	Mollisols	1542	22.12	22.80
Gigante Peninsula Experiment (Control/+P)	Miocene basalt	Oxisols and Inceptisols	484/662	0.57/16.7	0.71/32.2

*Note*: Soil P data for Gigante were collected several years prior to our sampling campaign and that soil P levels in the +P plots are increasing over time due to ongoing P additions; thus differences between controls and fertilised plots at the time of our sampling campaign were likely greater than those reflected in this Table.

^a^ Soil P data for sites comprising the natural fertility gradient represent the mean value of multiple surface (0–10 cm) soil cores taken at each site as described previously (Turner et al., 2018).

^b^ For the Gigante experimental plots, soil total P data (0–15 cm depth) are from Yavitt et al. (2011) and soil Mehlich and resin‐extractable P data (0–10 cm depth) are from Turner et al. (2013).

The Gigante Fertilization Experiment is located on Gigante Peninsula (9º06'31''N, 79º50'37''W), immediately south of Barro Colorado Island (Wright et al., 2011). Vegetation at Gigante is ~600‐year‐old forest (McMichael & Bush, 2024). Soils are low‐fertility Cambisols and Nitisols (FAO classification; Table 1). The experimental area covers 38 ha, with 32 40 m^2^ randomised experimental plots, most of which are buffered by a 40‐m distance from adjacent plots. Since 1998, fertilisers have been applied during the wet season. We considered only the P fertilisation (hereafter ‘+P’) treatment (*n* = 4) and unfertilised controls (*n* = 4). The +P treatment consisted of 50 kg P ha^−1^ year^−1^. Phosphorus was applied in the form of triple superphosphate (Ca(H_2_PO_4_)_2_.H_2_O), such that the P was accompanied by a substantial dose of Ca. This is not considered problematic because soils in central Panama typically have high Ca levels due to marine influences (Turner et al., 2013).

### 2.2 Sampling campaign

We used ground‐based flight‐intercept traps (FITs) to quantify the abundances of common, flight‐capable understorey Coleoptera families, along with Tullgren extractions of leaf litter to quantify the abundances of common epigeal Coleoptera families and of other common epigeal fauna. Sampling was carried out under Ministerio de Ambiente Scientific Permits SE/PO‐4‐18 and SE/PO‐5‐2019. The study did not require ethical approval.

Flight‐intercept traps were established in the 2019 Panamanian wet season (July/August) and were active for 14–20 days. We aimed to capture the seasonal peaks in abundance and diversity of Coleoptera in Panamanian forests (Erwin & Scott, 1980); however, tropical arthropods vary in their seasonality (Wolda, 1978, 1988), and some taxa peak outside our sampling period (e.g. in Panamanian lowland forests, Chrysomelidae abundance peaks in May/June; Richards & Windsor, 2007). Two FITs were installed at each of the 10 sites along the natural soil fertility gradient, with the two FITs located in likely flight paths, spaced at least 20 m apart and set at roughly orthogonal angles. At Gigante, only one FIT was installed per replicate plot, with FITs located close to the plot centres. Details of FIT construction are provided in Supporting Information Methods.

Litter samples for Tullgren extractions were collected in October 2019. Although later in the year than FIT sampling, October is well within the Panamanian wet season, and there are no obvious differences in rates of litterfall, quantities of standing litter biomass or concentrations of nutrients, including P, in litterfall between July and October (Wieder & Wright, 1995). Each sample consisted of three 1200 cm^2^ sub‐samples that were collected 5–10 m apart and combined. For sites on the natural soil P gradient, two samples were collected per plot, while at Gigante, only one sample was collected per plot. A separate set of litter samples was collected at each site (without sifting) to allow estimation of standing litter biomass. Details of litter sampling and extraction are provided in Supporting Information Methods.

### 2.3 Specimen sorting and counting

All Coleoptera specimens were sorted to family. Curculionidae, Scarabaeidae and Staphylinidae (together comprising 67 ± 3% of FIT catches and 54 ± 4% of Tullgren extracts) were then sorted to subfamily. Non‐Coleoptera in litter extracts were sorted to varying taxonomic levels, including phylum (Annelida, primarily Oligochaeta), class/subclass (Acari, Chilopoda, Diplopoda, Collembola), suborder (Blattodea and Isoptera), family (Formicidae) and Order for all other taxa. For simplicity, we refer to Coleoptera families and subfamilies as ‘families’ and broader arthropod taxa as ‘orders’. Coleoptera identification followed American Beetles volumes I and II (Arnett et al., 2002; Arnett & Thomas, 2014). Specimens are stored ethanol in taxon‐specific jars at the Smithsonian Tropical Research Institute's Tupper Facility in Panama City, Panama.

### 2.4 Data preparation and statistical analyses

Coleoptera abundances in FIT samples were standardised to a 14‐day period to account for minor differences in trapping duration among traps. For the 10 natural gradient sites, the abundances of Coleoptera in the two FITs at each site were summed to give one abundance estimate per taxon per site. Tullgren extracts were treated in the same way. Statistical analyses were carried out using R version 4.4.0 (R Core Team, 2024).

Our sampling intensity was low due to logistical challenges of carrying out concurrent trapping at 11 distributed locations. Although this is justified by our focus on common, relatively abundant taxa, and offset by our use of duplicate traps at each site (or at Gigante, by the small area of treatment plots), we evaluated our sampling sufficiency through rarefaction using the ‘*iNEXT*’ package (Hsieh et al., 2024; Watkins et al., 2017). Details and results of these rarefaction procedures are provided in the Supporting Information (Figures S5–S19). Completeness curves indicated excellent coverage of Coleoptera families in FITs and litter extracts and of fauna orders in litter extracts in almost all samples. Diversity curves showed that our sampling was sufficient for evaluating broad patterns in abundance and assemblage composition of common taxa: For the vast majority of samples, an extrapolated doubling of sampling load did not increase simulated taxonomic richness, Shannon diversity or Simpson diversity.

We evaluated the influence of soil P on Coleoptera and litter fauna assemblages through a combination of linear modelling, multivariate abundance modelling and non‐metric multidimensional scaling (NMDS) ordinations in combination with surface fitting techniques. We evaluated three indices of soil P, which are strongly correlated across the natural soil fertility gradient (Figure S1): resin‐extractable PO_4_^3−^, Mehlich‐extractable PO_4_^3−^ and total soil P. Results were qualitatively similar among these indices (Tables S1–S3); thus, for simplicity, we report results for total P (hereafter referred to as ‘soil P’).

To test our prediction that the size and diversity of arthropod assemblages should vary with soil P fertility, we examined linear associations between soil P and arthropod abundance and diversity using linear models. Here and elsewhere, soil P variables were natural log‐transformed and scaled prior to analyses. Total Coleoptera abundances in FITs and Tullgren extracts (with no transformations) were modelled using generalised linear models with negative binomial distributions, as appropriate for count data, with the ‘*MASS*’ package (Ripley et al., 2024). Estimated values of Coleoptera family Chao richness, Shannon diversity and Simpson diversity were extracted from our prior rarefaction analyses and analysed using linear models with normal distributions. Equivalent approaches were used to model the total abundance and diversity of fauna orders in Tullgren extracts.

Next, to test our prediction of variation in the strength of taxon‐specific abundance–soil P associations, we evaluated soil P as a predictor of the abundances of each Coleoptera family simultaneously using multivariate abundance analyses in the ‘*mvabund*’ package (Wang et al., 2022). Rare taxa, which we defined as families with <3 occurrences across the 10 sites, were excluded from multivariate analyses. Multivariate abundance models were based on negative binomial distributions and were specified to shrink correlation within the sample matrix. The latter specification is recommended when the number of taxa is large relative to the number of sites/plots because it accounts for correlations (i.e. non‐independence) among taxa while producing numerically stable test statistics. Taxon‐specific response coefficients (±95% confidence intervals) were then used to quantify the abundance responses of each taxon to soil P. An equivalent approach was used to model the abundances of litter fauna orders in Tullgren extracts. We also examined whether Coleoptera trophic guild abundances could be predicted based on soil P using multivariate abundance analysis. Here, Coleoptera families were assigned to trophic guilds (predators, fungivores, herbivores, saprophages, xylophages and ‘mixed’ feeders) based on prior assignations (Grimbacher & Stork, 2007) and previously observed feeding behaviours (Arnett et al., 2002; Arnett & Thomas, 2014).

To test our prediction of shifts in community composition from low‐fertility to high‐fertility sites, we used ‘*vegan*’ (Oksanen et al., 2024) to conduct NMDS analyses based on Bray–Curtis dissimilarities in two dimensions. We then used *vegan*'s ‘*ordisurf*’ function, which fits a smooth surface using a generalised additive regression model with penalised splines, to evaluate potential associations between the NMDS ordination site scores and soil P. This is equivalent to the environmental fitting method, but allows for non‐linear responses. *Ordisurf* analyses for the entire assemblage of litter fauna produced extreme *F*‐statistics (*F* > 10^6^) and consequently low *p* values; we regard these values as suspect and have excluded them from our results. More generally, while *ordisurf* does generate statistical *p* values, these are only approximate. Thus, we emphasise the descriptive utility of the *ordisurf* analysis over its inferential value.

To evaluate our prediction that the responses of arthropod assemblages to natural landscape‐level variation in soil fertility should be stronger than site‐level responses to experimental soil P fertilisation, we carried out a series of statistical analyses for the Gigante fertilisation experiment that were equivalent to those described above for the natural gradient, but applied to a completely randomised experiment. Here, the equivalent approach to the ‘*ordisurf*’ analysis was to use the ‘*envfit*’ command in *vegan*, with fertilisation treatment set as the environmental variable.

## 3 RESULTS

Ground‐based flight‐intercept trapping yielded 32,126 Coleoptera specimens spanning 68 families, whereas litter extraction yielded 1124 Coleoptera specimens spanning 28 families (Figure S4). The vast majority of Coleoptera specimens belonged to the Staphylinoidea (Staphylinidae + Ptiliidae). Beyond Coleoptera, 19 major taxa were found in litter, with 16,106 specimens extracted overall. Four taxa (Acari [48%], Formicidae [24%], Coleoptera [7%] and Collembola [6%] comprised the majority of specimens).

### 3.1 Abundance and composition of Coleoptera and litter fauna vary across landscape‐level gradients of soil fertility

**FIGURE 2:**
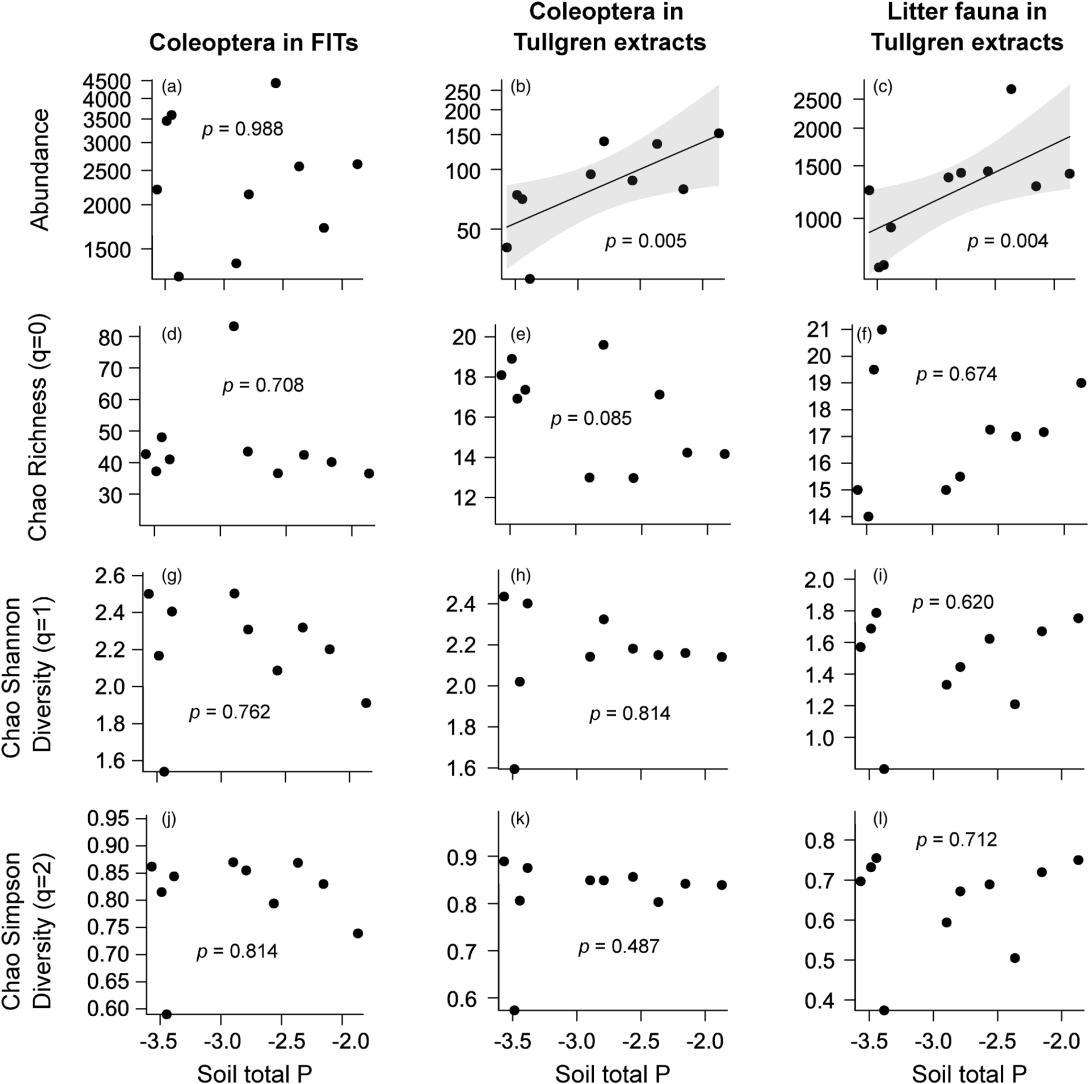
Landscape‐level relationships between soil total P (natural log‐transformed and scaled) and abundances of (a) Coleoptera caught in FITs, (b) Coleoptera extracted from litter, (c) all fauna extracted from litter, Chao richness of (d) Coleoptera families in FITs, (e) Coleoptera families extracted from litter, (f) orders of fauna extracted from litter, Chao Shannon diversity of (g) Coleoptera families in FITs, (h) Coleoptera families extracted from litter, (i) orders of fauna extracted from litter, Chao Simpson diversity of (j) Coleoptera families in FITs, (k) Coleoptera families extracted from litter, and (l) orders of fauna extracted from litter; *p* values indicate the significance of soil total P as a predictor of response variables, grey bands indicate 95% confidence intervals.

We found significant associations between natural variation in soil P and numerous characteristics of Coleoptera and litter fauna assemblages. Soil P was not a significant predictor of the total abundance of Coleoptera in FITs (Figure 2a); however, in Tullgren extracts, both the total abundance of Coleoptera and the total abundance of litter fauna were positively related to soil P (Figure 2b,c). Coleoptera abundances in Tullgren extracts increased almost threefold across our gradient, while total faunal abundance approximately doubled. No indices of diversity were associated with natural variation in soil P (Figure 2d–l).

**FIGURE 3:**
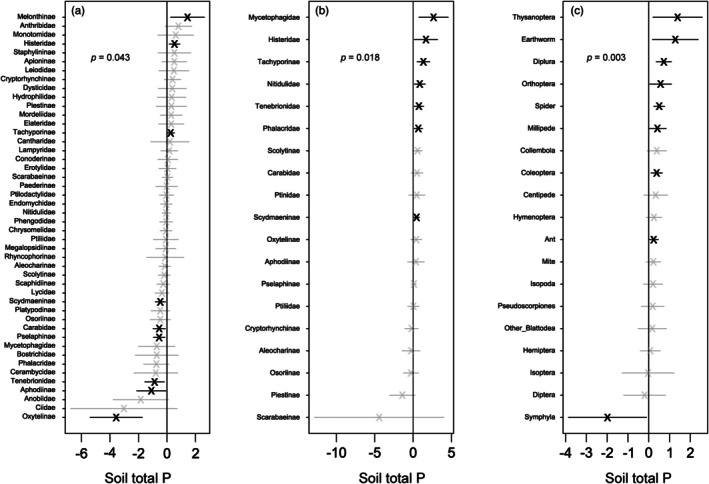
Taxon‐specific abundance responses to natural, landscape‐level variation in soil total P as derived from multivariate abundance models for (a) Coleoptera families in FITs, (b) Coleoptera families extracted from litter, and (c) arthropod orders extracted from litter; *p* values indicate the significance of soil total P (scaled and natural log‐transformed) as a predictor in multivariate abundance models (*n* = 10); bold points indicate a significant taxon‐specific response (positive or negative) to soil P.

Multivariate abundance analysis revealed that total Coleoptera abundance responses to soil P belied a diverse array of taxon‐specific responses, including strong negative associations, absences of association and strong positive associations (Figure 3a,b). For Coleoptera in FITs, soil P was a significant term in the multivariate abundance model (*p* = 0.043). Nine out of 46 Coleoptera families in FITs were sensitive to soil P (Figure 3a). These families represented 10–55% of the total number of Coleoptera specimens in FITs. Three families (Melonthinae, Histeridae and Tachyporinae) exhibited positive responses to soil P, and six families (Oxytelinae, Aphodiinae, Tenebrionidae, Pselaphinae, Carabidae and Scydmaeninae) responded negatively to soil P. For Coleoptera in Tullgren extracts, soil P was a significant term in our multivariate family abundance model (*p* = 0.018; Figure 3b). Seven of 19 litter‐extracted Coleoptera families were sensitive to soil P, representing 7%–86% of the total number of Coleoptera specimens. Unlike FITs, all of these responses were positive. Mycetophagidae exhibited the strongest response, followed by Histeridae, Tachyporinae, Nitidulidae, Tenebrionidae, Phalacridae and Scydmaeninae. Likewise, soil P was a strong predictor of the multivariate abundances of litter fauna orders beyond Coleoptera (Figure 3c). Nine of 19 orders exhibited strong associations with soil P, corresponding to 20%–69% of litter‐extracted fauna specimens, with eight of these orders showing positive associations. Only Symphyla exhibited a negative association with soil P.

**FIGURE 4:**
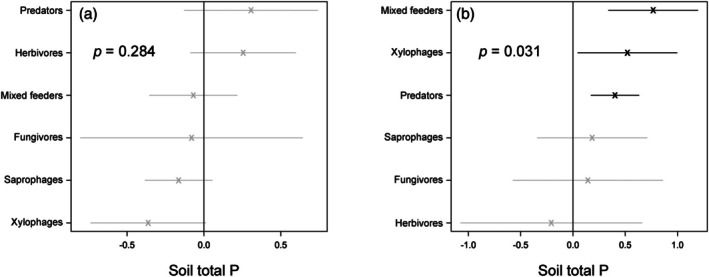
Trophic guild‐specific abundance responses of (a) flight‐intercept‐trapped Coleoptera and (b) litter‐extracted Coleoptera to natural, landscape‐level variation in soil total P as derived from multivariate abundance models; *p* values indicate the significance of soil total P in multivariate abundance models (*n* = 10); bold points indicate a significant response to soil P (positive or negative) for a given trophic guild.

Multivariate abundances on the level of Coleoptera trophic guild indicated soil P was not a strong, positive driver of the abundances of notionally fungivorous, herbivorous, predatory and mixed‐feeding Coleoptera in FITs (Figure 4a). In contrast, for Tullgren‐extracted specimens, the abundances of mixed‐feeding, xylophagous and predatory Coleoptera exhibited positive associations with soil P (*p* = 0.031; Figure 4b).

**FIGURE 5:**
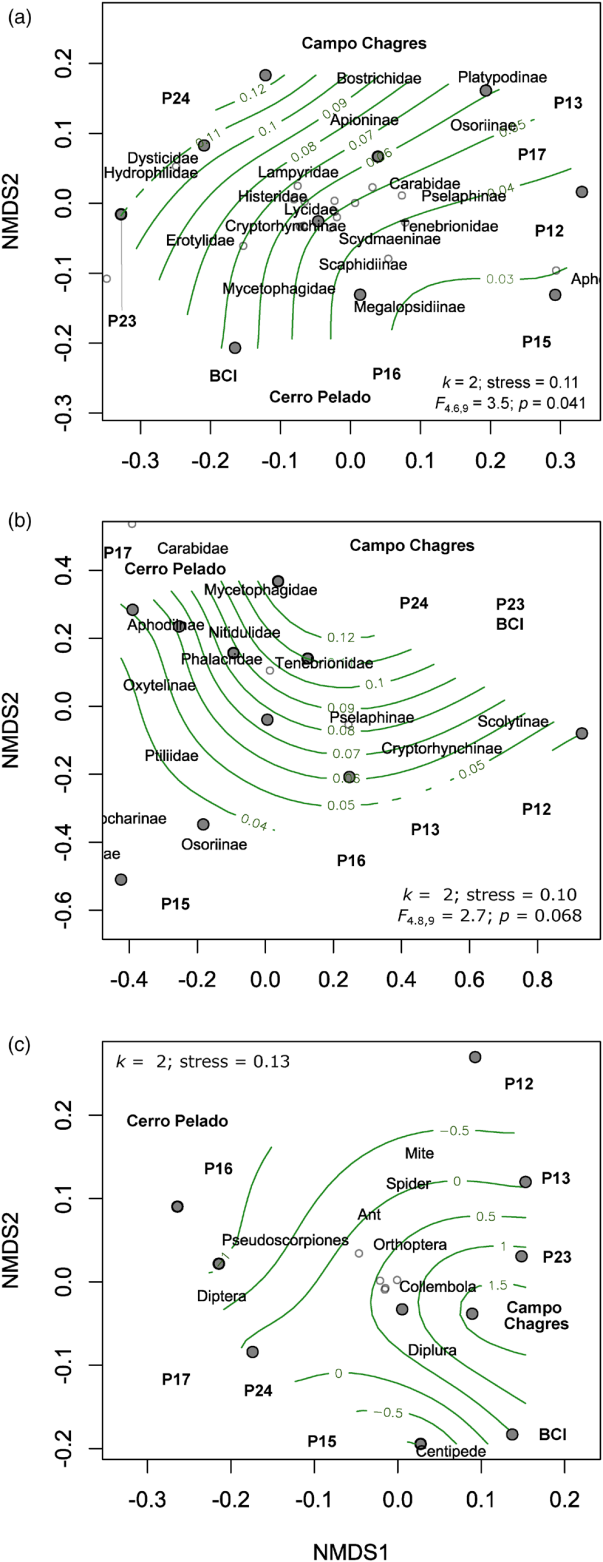
Non‐metric multidimensional scaling (NMDS) analyses and associated surface fitting of soil total P to NMDS site scores for (a) Coleoptera families in FITs, (b) Coleoptera families extracted from litter, and (c) fauna orders extracted from litter.

Non‐metric multidimensional scaling analyses and associated surface fitting analyses provided evidence that the suite of negative and positive P affinities of Coleoptera families and arthropod orders correspond to meaningful, albeit complex, trends in assemblage composition across the natural soil P gradient (Figure 5). The low approximate *p* values (<0.1) associated with the two‐dimensional smooth terms constitute weak‐to‐moderate evidence of a non‐linear association between total soil P and NMDS site scores for flight‐intercept‐trapped and litter‐extracted Coleoptera families.

### 3.2 Phosphorus‐fertilisation has limited effects on Coleoptera and litter fauna communities

**FIGURE 6:**
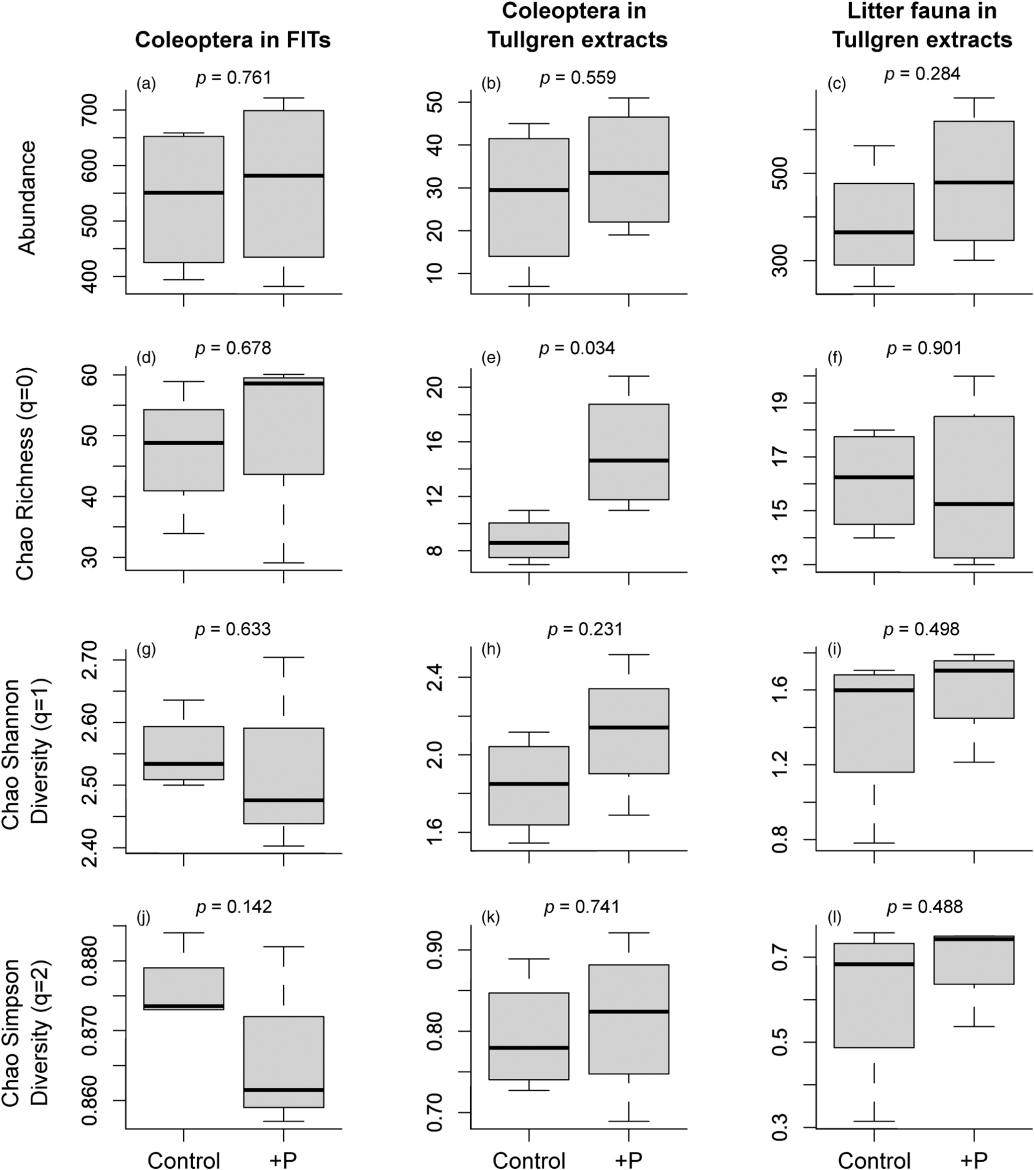
Effects of ~21 years of annual soil phosphorus (P) fertilisation on the abundance, richness and diversity of (a, d, g, j) Coleoptera families caught in FITs, (b, e, h, k) Coleoptera families extracted from litter, and (c, f, i, l) litter fauna orders extracted from litter (*n* = 4; *p* values indicate the significance of fertilisation as a source of variation in response variables).

Coleoptera abundance and family diversity were unaffected by P addition at Gigante regardless of sampling technique (Figure 6; Table S4). Coleoptera family Chao richness in Tullgren extracts was higher in P fertilised plots than unfertilised control plots, but this effect was not paralleled by FITs (Figure 6b,e). Experimental fertilisation had no effect on the multivariate abundances of Coleoptera families in FITs or litter extracts, and the same was true for arthropod orders in litter extracts (Table S5). Likewise, P addition did not affect the multivariate abundances of Coleoptera trophic guilds (Table S5). The insensitivity of Coleoptera and litter faunal abundances to P fertilisation at Gigante was reflected in NMDS ordinations and associated factor fitting analysis, which suggested little, if any, assemblage differentiation between control and fertilised plots (Figure S20).

## 4 DISCUSSION

Our survey of 10 lowland tropical forest sites spanning a wide soil fertility gradient revealed strong responses to soil P for (sub)family‐level abundance and composition of Coleoptera and Order‐level abundance and composition for epigeal arthropods generally. Prior work on this topic has hinted at a meaningful role for environmental P availability as a driver of the abundance and diversity of tropical arthropods. For example, previous work in the lowland forests of Costa Rica showed negative relationships between soil and/or litter C:P ratios and the density of pitfall‐trapped epigeal fauna (McGlynn et al., 2007), and research in Panama and Peru showed increasing abundance of Collembola and Isopoda with increasing litter P (Kaspari & Yanoviak, 2009). Likewise, litter P emerged as a key predictor of taxonomic richness of litter‐dwelling macro‐invertebrate assemblages in Sumatra (Jochum et al., 2017), and of the species richness of Blattarian assemblages in the Amazon Basin (Tarli et al., 2014). However, past studies have been equivocal and largely restricted to the ‘brown food web’, with a focus on litter P content as a driver of arthropod assembly. By contrast, we considered both epigeal (Tullgren‐extracted) and flight‐capable (FIT‐captured) Coleoptera at the level of subfamily, in addition to epigeal arthropods more broadly, and focused on soil P as an ultimate driver of responses that (presumably) exerts its influence via the quality, quantity or composition of plant‐derived basal resources. Thus, our study expands upon the scope of prior work and, by doing so, provides some of the strongest evidence to date that the abundance and assemblage composition of tropical arthropods is influenced by soil fertility, and specifically by soil P status.

In the case of flight‐intercept‐trapped Coleoptera, the orthogonality of absolute abundance and soil total P (Figure 2a) belied a diversity of strong, family‐specific responses (Figure 3a) that ranged from strongly positive, as in the case of Melonthinae (Scarabaeidae), to strongly negative, as in the case of Oxytelinae (Staphylinidae). Interestingly, this outcome parallels the trend evident for forest productivity across the broader soil P gradient in central Panama: community‐level forest productivity is largely stable across the ~300‐fold resin‐extractable soil PO_4_^3−^ gradient, and this stability is maintained by strong turnover of tree species that vary widely in their ‘P affinities’ (Condit et al., 2013; Turner et al., 2018). In this context, we find it worth noting that the proportion of Coleoptera families exhibiting significant P sensitivity (~20%) approaches the proportion of tree species exhibiting significant P affinities across the broader network of 72 forest inventory plots in central Panama (32%), particularly considering that we sampled a small subset of this plot network. We expect that species‐level Coleoptera P affinities could be similarly if not more widespread, especially given the moderate to strong plant host specificity common to tropical phytophagous Coleoptera species (Novotny & Basset, 2005; Ødegaard, 2006).

By contrast, the P affinities of Coleoptera families and invertebrate orders extracted from forest litter, where significant, were almost exclusively positive (Figure 3b,c), and the absolute abundance of Tullgren funnel‐extracted fauna was likewise positively related to soil P (Figure 2b,c). Thus, while there are instances where significant P affinities of Coleoptera families are consistent between FITs and Tullgren extracts (i.e. Histeridae and Tachyporinae), there are also some inconsistencies (i.e. Tenebrionidae and Scydmaeninae). Given the strong vertical stratification of Coleoptera species in tropical forests (Grimbacher & Stork, 2007), we suggest that discrepancies in family‐level P affinity between FIT catches and Tullgren extracts are due to differences in species composition between the two methods. These differences likely correspond to differences in feeding strategy and dispersal ability that render certain species more amenable to FITs than Tullgren extraction, and vice versa. For example, FIT catches likely have a higher proportion of large, flight‐capable species with strong dispersal abilities. Thus, we suggest that, although widespread, family‐level P affinities of Coleoptera are subject to various mediating factors (e.g. feeding strategy, dispersal ability and size) that limit our present ability to generalise about the P affinities of most families. Future work should give particular attention to such factors and other functional traits at family level or, ideally, species level, to better understand the causes of diverse P affinities in tropical forest Coleoptera taxa and arthropod taxa generally.

By combining a natural gradient of soil fertility with the experimental P manipulation at Gigante, our study hints at a potentially important role for vegetation composition as a proximal driver of tropical arthropod responses to landscape‐level soil P gradients. Indeed, despite the strong responses of arthropod assemblages to soil P across our natural gradient of soil fertility (Figures 2, 3, 4, 5), we observed almost no corresponding effects of P fertilisation treatments at Gigante Peninsula (Figure 6; Figure S20; Tables S4 and S5). This result contrasts with prior work at Gigante (Kaspari et al., 2017), which reported increases in the abundance of invertebrate fauna extracted from litter and soil samples in P‐fertilised plots. This difference may be due to the smaller sampling size in our study (*n* = 4) and the consequently lower power to detect small treatment effects. However, if landscape‐level variation in the P content and/or overall amounts of plant litter, stems, flowers, fruit and soil microbial biomass was entirely responsible for the trends we observed across the natural soil fertility gradient, it seems reasonable to expect similarly strong responses to chronic ecosystem P enrichment at Gigante. The fact that such responses were not observed is, in our view, a reasonably clear signal that neither resource P content (i.e. stoichiometric quality, as invoked by the structural shortage hypothesis) nor quantity (as invoked by the secondary productivity and ecosystem size hypotheses) are wholly responsible for the trends we observed across the natural fertility gradient. Thus, we are left with floristic composition, which varies tremendously across the central Panamanian soil P gradient (Umaña et al., 2021), but which was unaffected by P fertilisation at the time of our surveys (Wright et al., 2018).

Explanations other than floristic turnover across the landscape‐level soil fertility gradient do not fully justify the results we observed. For example, one might expect the positive trend between soil P and faunal abundance in Tullgren extracts to result from increasing litter quantity, but standing litter biomass was not associated with soil P across our sites (Pearson's *R* ^2^ < 0.01). Alternatively, some soil nutrient other than P might have caused the trends across the natural gradient. Available (i.e. Mehlich‐extractable) Ca was positively correlated with soil total P across our study sites (*R* ^2^ = 0.63). However, soils in central Panama are Ca‐rich due to marine influences (Turner et al., 2013), so arthropods are unlikely to be Ca‐limited, and the 50 kg ha^−1^ P additions at Gigante coincided with substantial additions of Ca, because P was applied as triple superphosphate [Ca(H_2_PO_4_)_2_.H_2_O]. Other nutrients were only moderately correlated (total N and KCl‐extractable NO_3_^−^) or uncorrelated with soil total P. Another candidate nutrient for confoundment is sodium, which is known to constrain the abundances of arthropods in the tropics and elsewhere (Kaspari, 2020; Kaspari et al., 2009). We lack soil sodium data for our sites, but we suggest that a strong, confounding correlation between soil P and Na is unlikely, given the general lack of correlation between P and most other basic cations other than Ca across our gradient. Another potential explanation is that fertilisation effects on soil P levels do not match the range of values provided by the natural soil fertility gradient. This is most relevant for soil total P, where values range from ~484 mg P kg soil^−1^ in control plots to ~662 mg P kg soil^−1^ in +P plots, compared with a range of 282–1542 mg P kg soil^−1^ across the natural gradient (Table 1). However, the range of values for Mehlich and resin‐extractable PO_4_^3−^–P at Gigante are well matched to the natural gradient (Table 1). Thus, given that (1) arthropod responses to natural variation in soil P levels were similar across the three P indices (albeit somewhat stronger for total P), (2) soil P levels in the +P fertilisation plots at Gigante are gradually increasing over time with repeated P additions subsequent to the P measurements reported in (Turner et al., 2013; Yavitt et al., 2011), and (3) arthropods do not eat soil P directly, but rather eat material derived from plants, microbes and other arthropods, all of which have been, or are likely to have been, enriched with P by P fertilisation at Gigante (Kaspari et al., 2008; Santiago et al., 2012; Schreeg et al., 2014; Turner & Wright, 2014; Wright et al., 2024; Wurzburger & Wright, 2015), we are confident that the effects of P fertilisation at Gigante are reasonably suitable for comparison with the natural soil fertility gradient. Perhaps the strongest alternative explanation is that sampling intensity across the natural gradient was 2.25‐fold greater than at Gigante. This is most likely to affect multivariate tests, rather than univariate abundance tests, due to potential biases associated with inadequately sampled, low‐abundance taxa. Our rarefaction analyses indicate low potential for such biases (Figures S5–S19). However, as an additional check, we randomly subsampled taxa from the natural gradient to match the corresponding, smaller number of taxa detected at Gigante, and repeated the multivariate abundance analyses. Results were qualitatively similar between these analyses, which gives us reasonable confidence in the adequacy of our sampling effort at Gigante, and our associated conclusions regarding the mediating effect of vegetation across the natural soil fertility gradient. Nevertheless, all of these potential alternative explanations should be considered when interpreting our findings.

It is well established that arthropod assemblages are influenced by floristic composition in the tropics and elsewhere (Basset et al., 2012; Schaffers et al., 2008; Zhang et al., 2016), and our study provides a reasonable, albeit indirect, indication that soil P might provide a template for this association. This insight opens the door to a rich suite of hypothetical biological mechanisms that support the coordination of plant and arthropod assemblages because the P affinities of tropical tree species have a basis in physiology (Umaña et al., 2021). High‐P affinity is associated with acquisitive resource strategies: community mean values of leaf mass area (LMA) and wood density decline as soil P increases across the central Panamanian soil P gradient, while the proportion of deciduous tree species increases (Umaña et al., 2021). By contrast, low‐P affinity is associated with conservative resource strategies (high LMA and wood density) and foliar longevity. Numerous leaf properties that inhibit herbivory and/or reduce palatability and decomposability, such as structural resistance/toughness, mesophyll cell wall thickness and C to nutrient ratios (Onoda et al., 2011, 2017; Westbrook et al., 2011), are usually positively correlated with LMA, which itself tends to be negatively correlated with herbivory and decomposition in tropical forests (Bakker et al., 2011; Kurokawa & Nakashizuka, 2008). Conservative plant resource strategies also include synthesis of metabolites that deter herbivores and which can have subsequent impacts on the brown food web (Coley & Barone, 1996; Coq et al., 2010). Thus, the conservative–acquisitive trait continuum of tropical trees likely translates to a low‐quality–high‐quality resource continuum for tropical heterotrophs. Under this speculative scenario, landscape‐scale gradients of soil P fertility regulate the relative quantity of high‐quality plant‐derived basal resources at a given location by driving spatial turnover of tropical tree species along a conservative–acquisitive trait continuum. This turnover then drives a wide variety of arthropod taxon‐specific responses to the underlying soil P gradient, from negative to positive, depending on the resource preferences and functional traits of the taxon in question, which in turn drives spatial variation in overall arthropod assemblage composition. In this way, the diverse geological history of the Panamanian Isthmus propagates throughout ecosystems and across landscapes by giving rise to a variety of soil types that shape floristic composition and, in turn, the abundances and assemblage composition of tropical forest arthropods.

Together, our results demonstrate the meaningful role of soil fertility in shaping understorey arthropod assemblages across lowland tropical forest landscapes. A range of P affinities can exist within and among arthropod orders, which can lead to wholesale shifts in arthropod assemblage composition across soil fertility gradients. We speculate that the prevalence of arthropod P affinities is related to the documented range of P affinities among tropical tree species (Condit et al., 2013; Turner et al., 2018), which gives rise to a landscape‐scale gradient of basal resource quality (Umaña et al., 2021), and that variation in P affinity among arthropod taxa is driven by functional traits, such as body size, growth rate, flight/dispersal ability and trophic habit. Evaluation of these hypotheses should involve long‐term monitoring of arthropods across natural and experimental soil fertility gradients and at sites with experimentally altered tree composition, assessment of insect herbivore performance on low‐P and high‐P specialist plant species in common gardens, and detailed characterisations of the functional trait composition of arthropod assemblages to inform ‘fourth corner’ species distribution models (Brown et al., 2014). Through these approaches, we can begin to resolve the many remaining uncertainties about the role of P and other limiting nutrients in driving soil–plant–animal interactions within and across tropical landscapes.

## AUTHOR CONTRIBUTIONS

Orpheus M. Butler and Donald Windsor conceived the ideas and designed methodology. Orpheus M. Butler, Vanessa Sanchez and Kara J. Simpson collected the data, with support from Donald Windsor; Orpheus M. Butler analysed the data and led the writing of the manuscript. All authors contributed critically to the drafts and gave final approval for publication.

## CONFLICT OF INTEREST STATEMENT

The authors have no conflict of interest to declare.

## Supporting information


**Figure S1.** Correlations among three indices of surface soil (0–10 cm depth) phosphorus (P) content across a natural, landscape‐scale gradient of soil fertility in the lowland tropical forests of central Panama.
**Figure S2.** Scatterplots demonstrating orthogonality of (a) modelled annual precipitation and (b) site elevation with respect to surface soil (0–10 cm) total phosphorus content across a natural, landscape‐scale gradient of soil fertility in the lowland tropical forests of central Panama.
**Figure S3.** Timing and duration of flight‐intercept trap establishment and activation for each of the ten study plots and the Gigante fertilisation experiment in. Green cells represent days during which FITs were active.
**Figure S4.** Pie charts showing the abundances of (a) Coleoptera families, (b) Staphylinidae subfamilies, and (c) Curculionidae subfamilies caught in flight‐intercept traps and (d) Coleoptera families, (e) Staphylinidae subfamilies, and (f) Curculionidae subfamilies extracted from litter 252 samples via Tullgren funnels.
**Figure S5.** Sample completeness curves (with 95% confidence intervals as shaded areas) for Coleoptera families caught in ground‐based flight‐intercept traps in Panamanian lowland tropical forests over a standardised two‐week period.
**Figure S6.** Sample completeness curves (with 95% confidence intervals as shaded areas) for Coleoptera families extracted from Panamanian lowland tropical forest litter.
**Figure S7.** Sample completeness curves (with 95% confidence intervals as shaded areas) for litter fauna orders extracted from Panamanian lowland tropical forest litter.
**Figure S8.** Sample size‐based rarefaction and extrapolation sampling curves (with 95% confidence intervals as shaded areas) for Coleoptera families caught in ground‐based flight‐intercept traps in Panamanian lowland tropical forests over a standardised two‐week period. Samples were collected across a landscape‐scale network of ten sites that spanned a wide, natural gradient of soil phosphorus fertility.
**Figure S9.** Sample size‐based rarefaction and extrapolation sampling curves (with 95% confidence intervals as shaded areas) for Coleoptera families extracted from Panamanian lowland tropical forest litter.
**Figure S10.** Sample size‐based rarefaction and extrapolation sampling curves (with 95% confidence intervals as shaded areas) for litter fauna orders extracted from Panamanian lowland tropical forest litter.
**Figure S11.** Sample completeness curves (with 95% confidence intervals as shaded areas) for Coleoptera families caught in ground‐based flight‐intercept traps in the control and +P treatments at the Gigante peninsula forest fertilisation experiment in central Panama.
**Figure S12.** Sample completeness curves (with 95% confidence intervals as shaded areas) for Coleoptera families extracted from forest litter collected from the control and +P treatments at the Gigante peninsula forest fertilisation experiment in central Panama.
**Figure S13.** Sample completeness curves (with 95% confidence intervals as shaded areas) for fauna orders extracted from forest litter collected from the control and +P treatments at the Gigante peninsula forest fertilisation experiment in central Panama.
**Figure S14.** Sample size‐based rarefaction and extrapolation sampling curves (with 95% confidence intervals as shaded areas) for Coleoptera family richness (*q* = 0) in ground‐based flight‐intercept traps installed in the control and +P treatments of the Gigante peninsula forest fertilisation experiment in central Panama.
**Figure S15.** Sample size‐based rarefaction and extrapolation sampling curves (with 95% confidence intervals as shaded areas) for Coleoptera family Shannon diversity (*q* = 1) and Simpson diversity (*q* = 2) in ground‐based flight‐intercept traps installed in the control and +P treatments of the Gigante peninsula forest fertilisation experiment in central Panama.
**Figure S16.** Sample size‐based rarefaction and extrapolation sampling curves (with 95% confidence intervals as shaded areas) for Coleoptera family richness (*q* = 0) in extracts of forest litter collected from the control and +P treatments of the Gigante peninsula forest fertilisation experiment in central Panama.
**Figure S17.** Sample size‐based rarefaction and extrapolation sampling curves (with 95% confidence intervals as shaded areas) for Coleoptera family Shannon diversity (*q* = 1) and Simpson diversity (*q* = 2) in extracts of forest litter collected from the control and +P treatments of the Gigante peninsula forest fertilisation experiment in central Panama.
**Figure S18.** Sample size‐based rarefaction and extrapolation sampling curves (with 95% confidence intervals as shaded areas) for order‐level richness (*q* = 0) of fauna extracted from forest litter collected from the control and +P treatments of the Gigante peninsula forest fertilisation experiment in central Panama.
**Figure S19.** Sample size‐based rarefaction and extrapolation sampling curves (with 95% confidence intervals as shaded areas) for order‐level Shannon diversity (*q* = 1) and Simpson diversity (*q* = 2) of fauna extracted from forest litter collected from the control and +P treatments of the Gigante peninsula forest fertilisation experiment in central Panama.
**Figure S20.** Non‐metric multidimensional scaling [NMDS] and associated factor fitting analysis examining relationships between soil P fertilisation treatment and the assemblage composition of (a) Coleoptera families caught in flight‐intercept traps, (b) Coleoptera families extracted from forest litter, and (c) arthropods orders extracted from forest litter in the lowland tropical forest of Gigante peninsula in central Panama.
**Table S1.** Summary of outputs of linear models used to evaluate the significance of soil phosphorus (P) indices as predictors of understorey arthropod abundance and diversity across a landscape‐scale gradient of soil fertility in the lowland tropical forests of central Panama.
**Table S2.** Summary of outputs of multivariate abundance models used to evaluate the significance of soil phosphorus (P) indices as predictors of the taxon‐specific abundances understorey arthropods across a landscape‐scale gradient of soil fertility in the lowland tropical forests of central Panama.
**Table S3.** Summary of outputs of ‘ordisurf’ surface fitting analysis used to evaluate the associations between the composition of understorey arthropod assemblages and soil phosphorus (P) indices across a landscape‐scale gradient of soil fertility in the lowland tropical forests of central Panama.
**Table S4.** Summary of results of one‐way ANOVAs testing the effects of long‐term phosphorus fertilisation on the abundance and diversity of understorey arthropods in the lowland tropical forests of Gigante Peninsula, central Panama.
**Table S5.** Summary of results of multivariate abundance analyses testing the effects of long‐term phosphorus fertilisation on the taxon‐specific abundances of understorey arthropods in the lowland tropical forest of Gigante Peninsula, central Panama.

## Data Availability

Data supporting the results reported in this study are available from the Zenodo repository: https://doi.org/10.5281/zenodo.14963480 (Butler et al., 2025).
