# Supplementary material for: Arthropod abundances track soil fertility across a lowland tropical forest landscape
Source: J Anim Ecol. 2025 May 23;94(7):1383–97. doi: 10.1111/1365-2656.70060 (PMC12214449; doi:10.1111/1365-2656.70060)
Supplement: Supplementary file 1 — Figure S1. Correlations among three indices of surface soil (0–10 cm depth) phosphorus (P) content across a natural, landscape‐scale gradient of soil fertility in the lowland tropical forests of central Panama. Figure S2. Scatterplots demonstrating orthogonality of (a) modelled annual precipitation and (b) site elevation with respect to surface soil (0–10 cm) total phosphorus content across a natural, landscape‐scale gradient of soil fertility in the lowland tropical forests of central Panama. Figure S3. Timing and duration of flight‐intercept trap establishment and activation for each of the ten study plots and the Gigante fertilisation experiment in. Green cells represent days during which FITs were active. Figure S4. Pie charts showing the abundances of (a) Coleoptera families, (b) Staphylinidae subfamilies, and (c) Curculionidae subfamilies caught in flight‐intercept traps and (d) Coleoptera families, (e) Staphylinidae subfamilies, and (f) Curculionidae subfamilies extracted from litter 252 samples via Tullgren funnels. Figure S5. Sample completeness curves (with 95% confidence intervals as shaded areas) for Coleoptera families caught in ground‐based flight‐intercept traps in Panamanian lowland tropical forests over a standardised two‐week period. Figure S6. Sample completeness curves (with 95% confidence intervals as shaded areas) for Coleoptera families extracted from Panamanian lowland tropical forest litter. Figure S7. Sample completeness curves (with 95% confidence intervals as shaded areas) for litter fauna orders extracted from Panamanian lowland tropical forest litter. Figure S8. Sample size‐based rarefaction and extrapolation sampling curves (with 95% confidence intervals as shaded areas) for Coleoptera families caught in ground‐based flight‐intercept traps in Panamanian lowland tropical forests over a standardised two‐week period. Samples were collected across a landscape‐scale network of ten sites that spanned a wide, natural gradient of soil phosphor [file JANE-94-1383-s001.pdf]

# Supporting Information

## Supplementary Methods

Flight-intercept trapping

Ground-based flight-intercept traps (FITs) were deployed at each site during the Panamanian wet season. Each FIT consisted of a 90 cm wide × 200 cm tall lightweight, black mesh suspended vertically from ground level and pinned down with steel pegs. Six aluminium trays (25 cm wide × 30 cm long × 6 cm deep) were arranged on the ground on both sides of the base of each mesh screen. These trays were each filled with ~800 ml of 40% propylene glycol preservative solution along with 2-3 drops of phosphate-free dish-washing detergent to break the surface tension of the propylene glycol solution. A tarpaulin was suspended above each mesh screen and its associated trays to prevent rain from falling into the trays.

We made substantial effort to randomise the order of flight-intercept trapping period with respect to the soil P gradient, and to ensure that seasonal effects did not influence our comparison of the natural gradient to the Gigante fertilisation experiment. The timing of our sampling campaign is shown in Figure S3.

Litter collection and arthropod extraction

Forest litter was collected at each site towards the end of the Panamanian wet season. Each sample (two samples per site for each natural gradient site and one sample per plot for each of the experimental plots at Gigante) was comprised of three sub-samples that were collected within a ~20 $m^2$ area and combined. Each sub-sample was collected by quickly and carefully scooping litter material into a plastic pan (24 cm wide) along a length of 50 cm, giving a sampling area of 1200 $cm^2$ per sub-sample. Litter material in the pan was then immediately sifted through nylon mesh (2 cm aperture) and the siftates were retained in a cotton cloth bag and taken back to the laboratory for same-day extraction of litter fauna. To prevent the fine litter material from falling directly through the funnel opening and into our collection jar, we placed a disc of 5 mm aperture wire mesh at the bottom of each funnel. Thus, some larger invertebrates (e.g., those with width >5 mm) will have been excluded from our extraction. Litter fauna were extracted into jars containing 70% ethanol solution under 25 W incandescent bulbs over three days, after which time we observed few, if any, additional specimens with an additional day of extraction.

Rarefaction analyses

Specifically, for each of the ten sites that comprise the natural fertility gradient and each replicate plot at Gigante, we generated sample size-based completeness curves, in which sample coverage (the estimated proportion of individuals in an assemblage represented by the families sampled (Chao and Jost 2012)) is plotted against the standardised number of individuals in each sample, and diversity curves in which the diversity of Coleoptera families and litter fauna orders is plotted against the number of individuals in each sample. Ninety-five percent confidence intervals were generated for these curves based on 50 bootstrap iterations. Results of rarefaction analyses are provided in the Supplementary Material (Figures S4–18).

## Supplementary Results

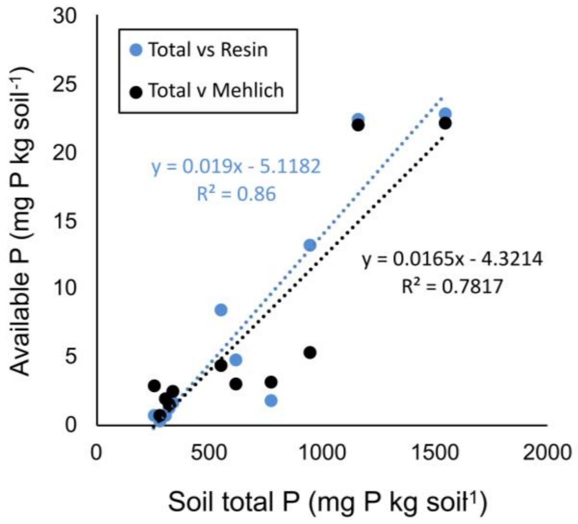


**Fig. S1.** Correlations among three indices of surface soil (0–10 cm depth) phosphorus (P) content across a natural, landscape-scale gradient of soil fertility in the lowland tropical forests of central Panama.

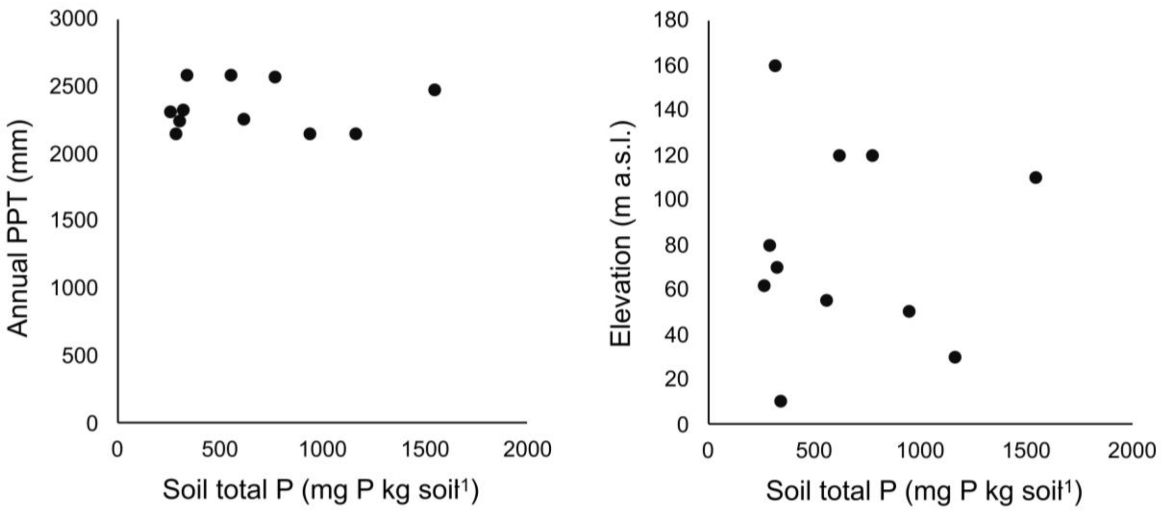


**Fig. S2.** Scatterplots demonstrating orthogonality of (a) modelled annual precipitation and (b) site elevation with respect to surface soil (0–10 cm) total phosphorus content across a natural, landscape-scale gradient of soil fertility in the lowland tropical forests of central Panama.

| Month | | July 2019 | | | | | | | | | | | | | | | | | | | | | | | | | | | | August 2019 | | | | | | | | | | |
|---|---|---|---|---|---|---|---|---|---|---|---|---|---|---|---|---|---|---|---|---|---|---|---|---|---|---|---|---|---|---|---|---|---|---|---|---|---|---|---|---|
| Date | | 4 | 5 | 6 | 7 | 8 | 9 | 10 | 11 | 12 | 13 | 14 | 15 | 16 | 17 | 18 | 19 | 20 | 21 | 22 | 23 | 24 | 25 | 26 | 27 | 28 | 29 | 30 | 31 | 1 | 2 | 3 | 4 | 5 | 6 | 7 | 8 | 9 | 10 | 11 |
| | Cerro Pelado | | | | | FITs activated | | | | Collection | | | FITS re-activated | | | | | | | | Collection | | | | | | | | | | | | | | | | | | | |
| | P16 | | | | | FITs activated | | | | | | | | | | | | | | | | | Collection | | | | | | | | | | | | | | | | | |
| | P15 | | | | | | | | | | | | | | | FITs activated | | | | | | | | | | | | | Collection | | | | | | | | | | | |
| | P12 | | | | | | FITs activated | | | | | | | Collection | | | FITs re-activated | | | | | Collection | | | | | | | | | | | | | | | | | | |
| | BCI | | | | | | | | | | | | | | | | FITs activated | | | | | Collection | | | | | | | | Collection | | | | | | | | | | |
| | P17 | | | | | FITs activated | | | | | | | | | | | | | | | | | Collection | | | | | | | | | | | | | | | | | |
| | P13 | | | | | | FITs activated | | | | | | | Collection | | | FITs re-activated | | | | | Collection | | | | | | | | | | | | | | | | | | |
| | P24 | | | | | | | | | | | | | | | | | | | | FIT installed and activated | | | | | | | | | | Collection | | | | | | Collection | | | |
| Site | P23 | FITs activated | | | | | | | Collection | | | | | | | | | | | | Collection | | | | | | | | | | | | | | | | | | | |
| | Campo Chagres | | | | | | | | | | | | FITs activated | | | | | | | | Collection | | | | | | | | Collection | | | | | | | | | | | |
| | Gigante Control - 1 | | | | | | | FIT activated | | | | | | | Collection | | | | | | | | | | | | Collection | | | | | | | | | | | | | |
| | Gigante Control - 2 | | | | | | | FIT activated | | | | | | | Collection | | | | | | | | | | | | Collection | | | | | | | | | | | | | |
| | Gigante Control - 3 | | | | | | | FIT activated | | | | | | | Collection | | | | | | | | | | | | Collection | | | | | | | | | | | | | |
| | Gigante Control - 4 | | | | | | | FIT activated | | | | | | | Collection | | | | | | | | | | | | Collection | | | | | | | | | | | | | |
| | Gigante +P - 1 | | | | | | | FIT activated | | | | | | | Collection | | | | | | | | | | | | Collection | | | | | | | | | | | | | |
| | Gigante +P - 2 | | | | | | | FIT activated | | | | | | | Collection | | | | | | | | | | | | Collection | | | | | | | | | | | | | |
| | Gigante +P - 3 | | | | | | | FIT activated | | | | | | | Collection | | | | | | | | | | | | Collection | | | | | | | | | | | | | |
| | Gigante +P - 4 | | | | | | | FIT activated | | | | | | | Collection | | | | | | | | | | | | Collection | | | | | | | | | | | | | |

**Figure S3**. Timing and duration of flight-intercept trap establishment and activation for each of the ten study plots and the Gigante fertilisation experiment in. Green cells represent days during which FITs were active.

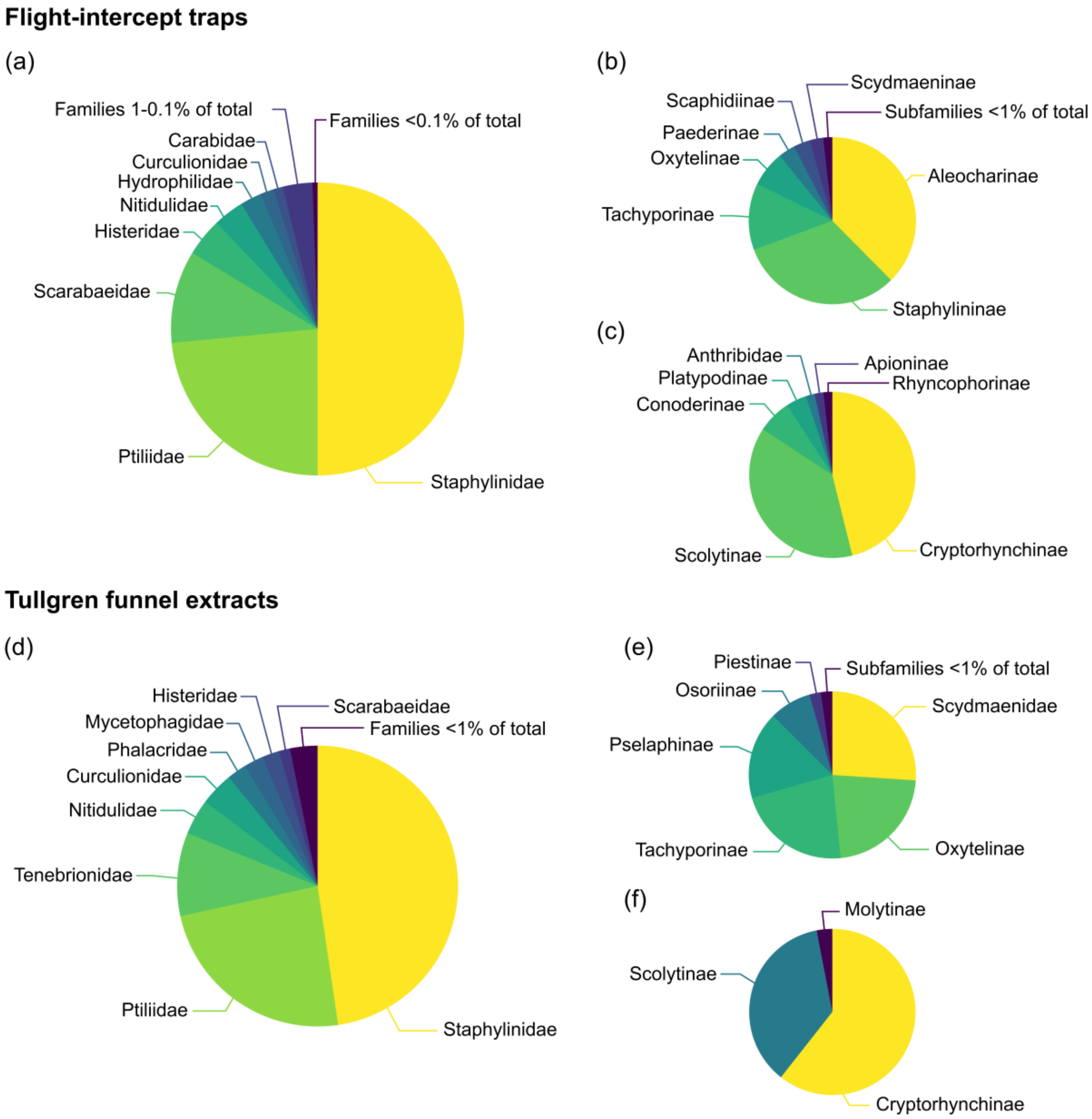


**Figure S4.** Pie charts showing the abundances of (a) Coleoptera families, (b) Staphylinidae subfamilies, and (c) Curculionidae subfamilies caught in flight-intercept traps and (d) Coleoptera families, (e) Staphylinidae subfamilies, and (f) Curculionidae subfamilies extracted from litter 252 samples via Tullgren funnels.

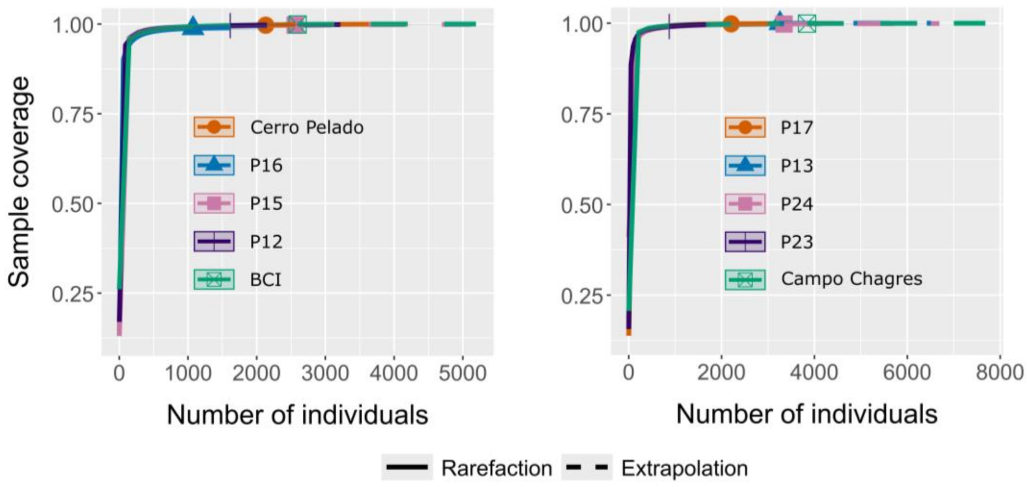


**Figure S5.** Sample completeness curves (with 95% confidence intervals as shaded areas) for Coleoptera families caught in ground-based flight-intercept traps in Panamanian lowland tropical forests over a standardised two-week period. Samples were collected across a landscape-scale network of ten sites that spanned a wide, natural gradient of soil phosphorus fertility. The ten plots are presented across two separate panels for ease of visualisation.

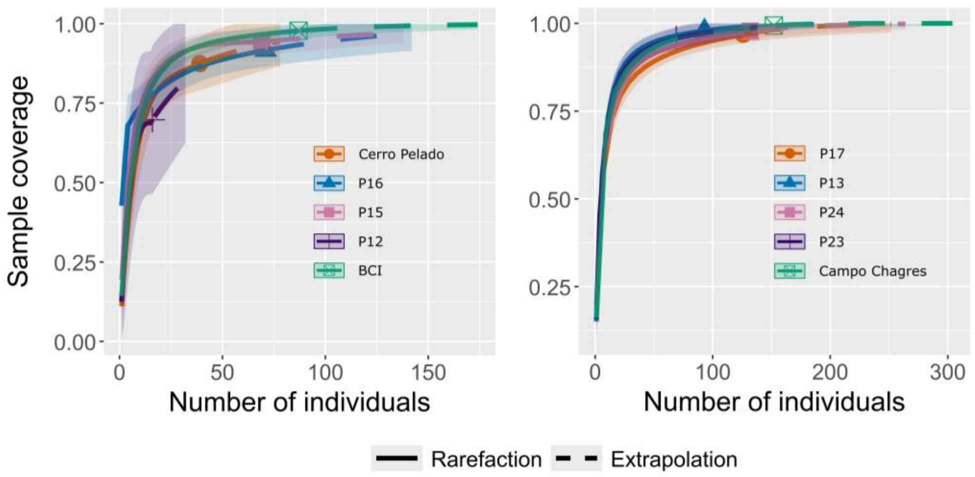


**Figure S6.** Sample completeness curves (with 95% confidence intervals as shaded areas) for Coleoptera families extracted from Panamanian lowland tropical forest litter. Samples were collected across a landscape-scale network of ten sites that spanned a wide, natural gradient of soil phosphorus fertility. The ten plots are presented across two separate panels for ease of visualisation.

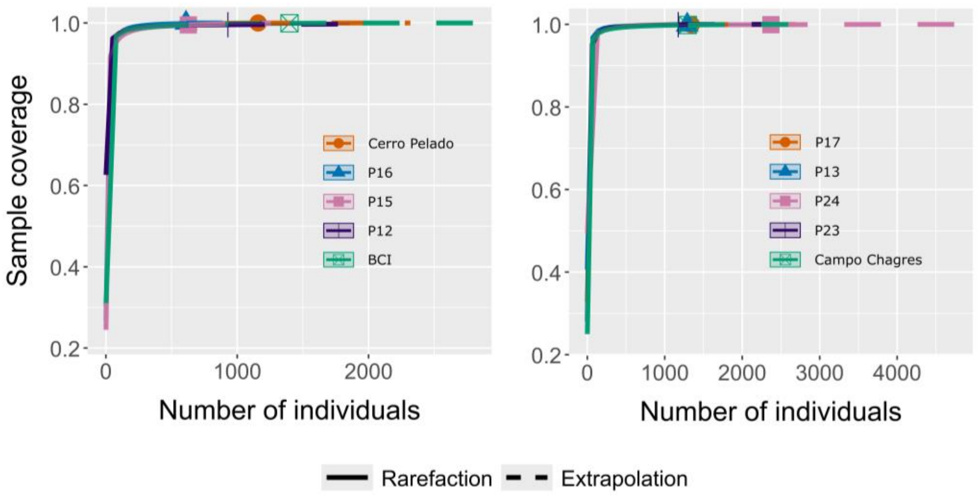


**Figure S7.** Sample completeness curves (with 95% confidence intervals as shaded areas) for litter fauna orders extracted from Panamanian lowland tropical forest litter. Samples were collected across a landscape-scale network of ten sites that spanned a wide, natural gradient of soil phosphorus fertility. The ten plots are presented across two separate panels for ease of visualisation.

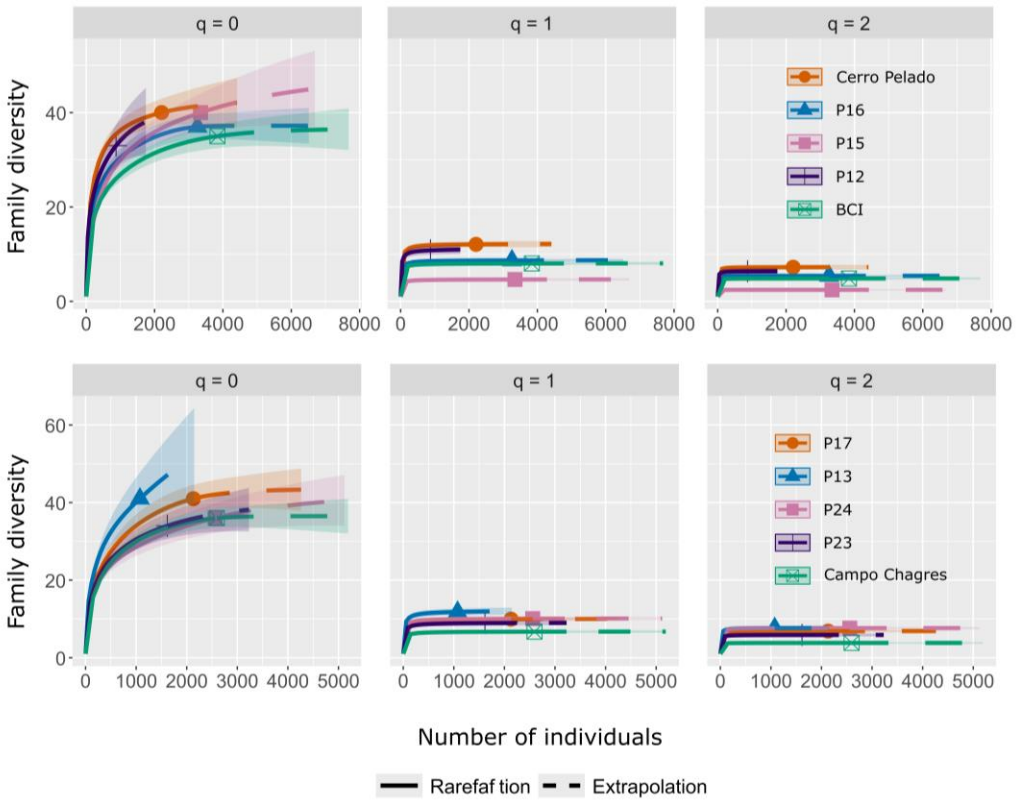


**Figure S8.** Sample size-based rarefaction and extrapolation sampling curves (with 95% confidence intervals as shaded areas) for Coleoptera families caught in ground-based flight-intercept traps in Panamanian lowland tropical forests over a standardised two-week period. Samples were collected across a landscape-scale network of ten sites that spanned a wide, natural gradient of soil phosphorus fertility. Curves for the three Chao diversity orders are plotted separately: q = 0 (richness, i.e., number of families), q = 1 (Shannon diversity of families), q = 2 (Simpson diversity of families). Legend in top right panel applies to all upper row panels, legend in bottom right panel applies to all lower row panels. The ten plots are presented across two separate panels (upper and lower panels in each column) for ease of visualisation.

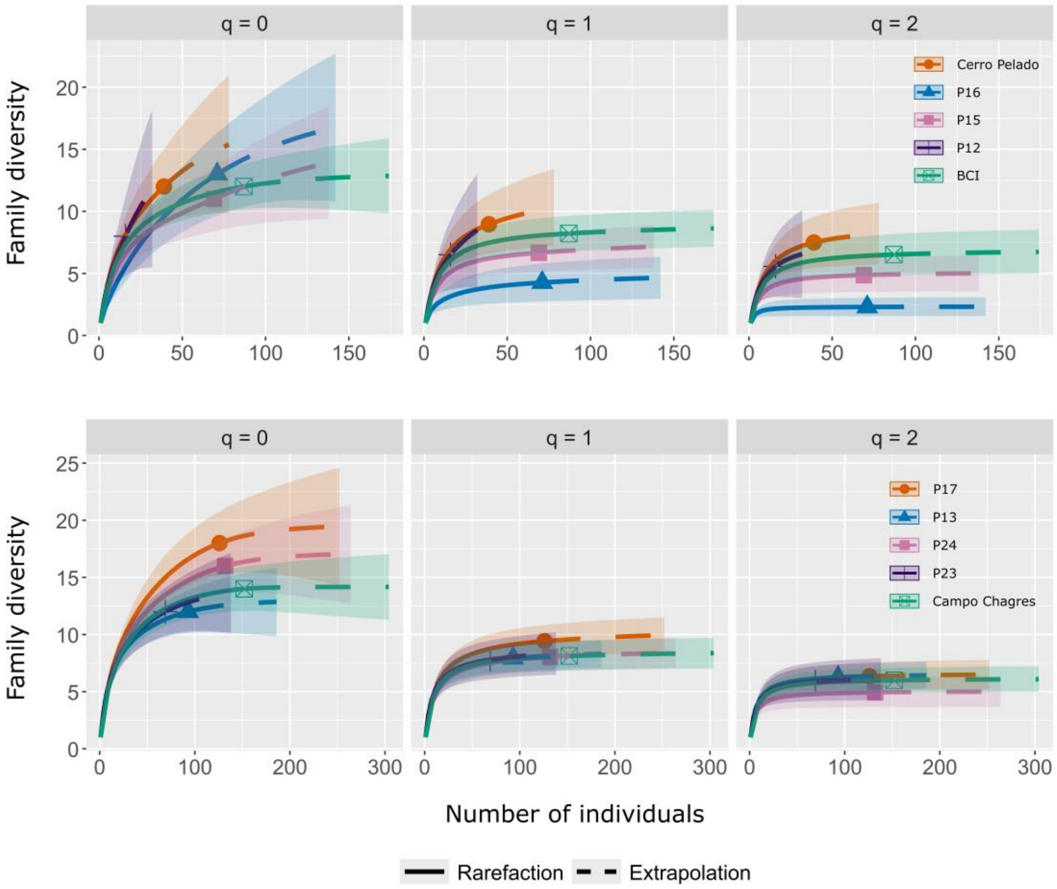


**Figure S9.** Sample size-based rarefaction and extrapolation sampling curves (with 95% confidence intervals as shaded areas) for Coleoptera families extracted from Panamanian lowland tropical forest litter. Samples were collected across a landscape-scale network of ten sites that spanned a wide, natural gradient of soil phosphorus fertility. Curves for the three Chao diversity orders are plotted separately: q = 0 (richness, i.e., number of families), q = 1 (Shannon diversity of families), q = 2 (Simpson diversity of families). Legend in top right panel applies to all upper row panels, legend in bottom right panel applies to all lower row panels. The ten plots are presented across two separate panels (upper and lower panels in each column) for ease of visualisation.

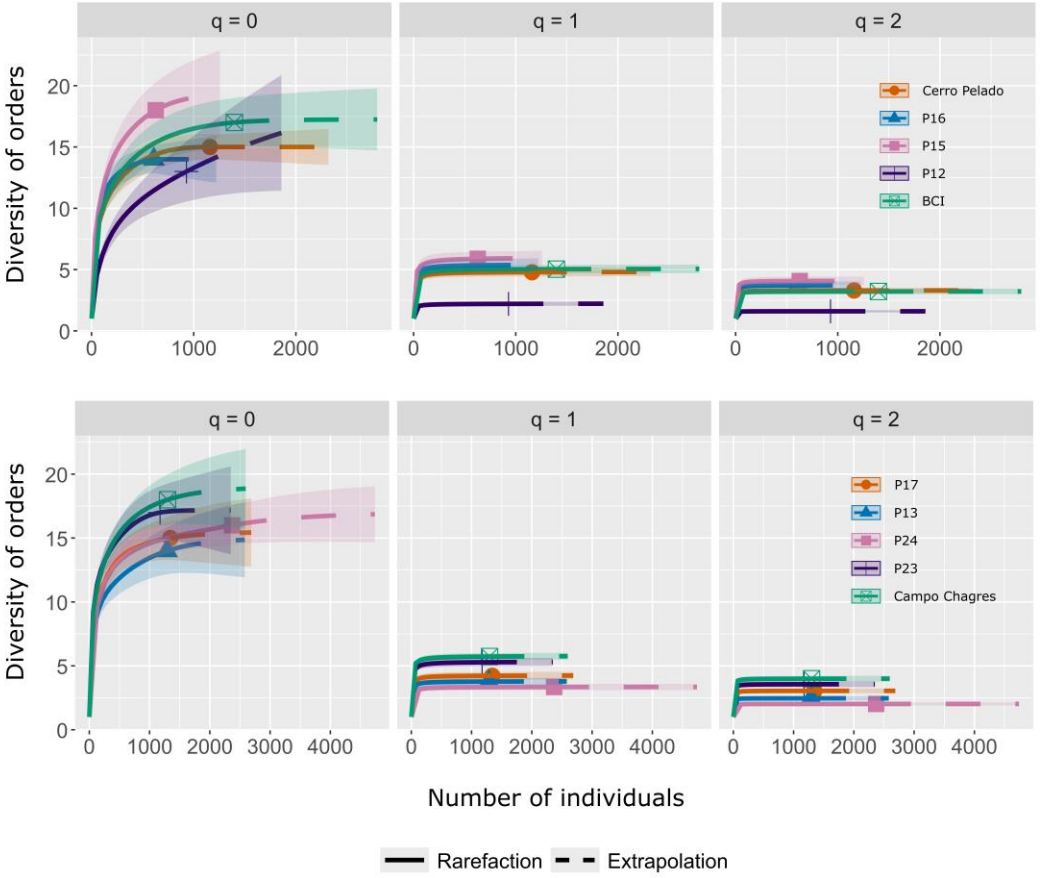


**Figure S10.** Sample size-based rarefaction and extrapolation sampling curves (with 95% confidence intervals as shaded areas) for litter fauna orders extracted from Panamanian lowland tropical forest litter. Samples were collected across a landscape-scale network of ten sites that spanned a wide, natural gradient of soil phosphorus fertility. Curves for the three Chao diversity orders are plotted separately: q = 0 (richness, i.e., number of orders), q = 1 (Shannon diversity of orders), q = 2 (Simpson diversity of orders). Legend in top right panel applies to all upper row panels, legend in bottom right panel applies to all lower row panels. The ten plots are presented across two separate panels (upper and lower panels in each column) for ease of visualisation.

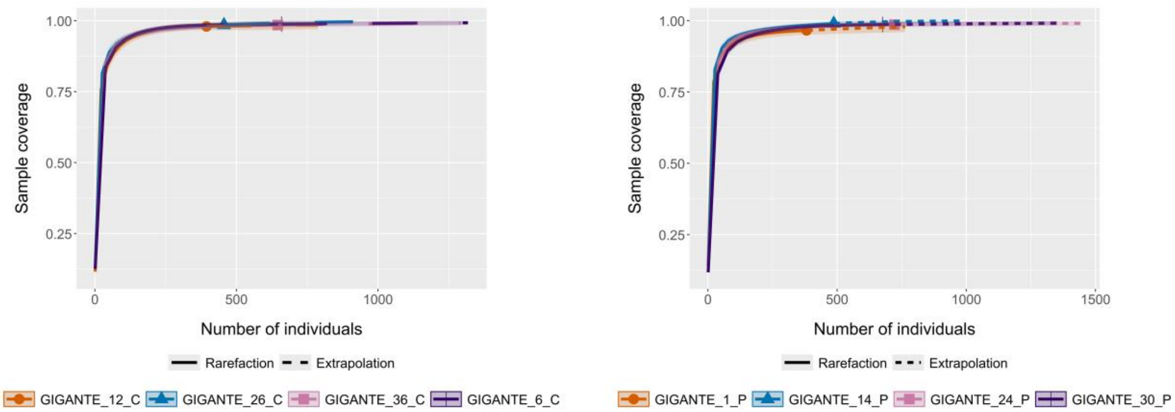


**Figure S11.** Sample completeness curves (with 95% confidence intervals as shaded areas) for Coleoptera families caught in ground-based flight-intercept traps in the control and +P treatments at the Gigante peninsula forest fertilisation experiment in central Panama. The eight plots are presented across two separate panels for ease of visualisation; control plots are presented in the panel on the left, fertilised plots are presented in the panel on the right.

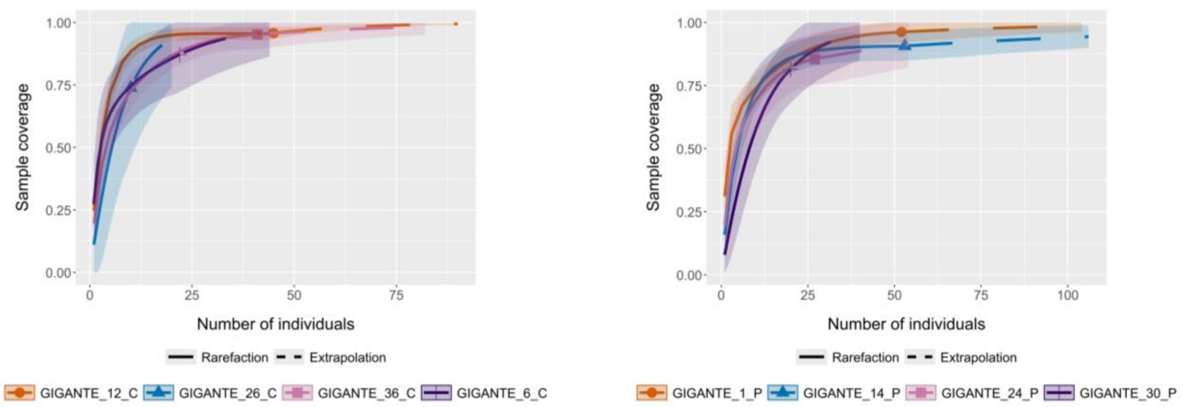


**Figure S12.** Sample completeness curves (with 95% confidence intervals as shaded areas) for Coleoptera families extracted from forest litter collected from the control and +P treatments at the Gigante peninsula forest fertilisation experiment in central Panama. The eight plots are presented across two separate panels for ease of visualisation; control plots are presented in the panel on the left, fertilised plots are presented in the panel on the right.

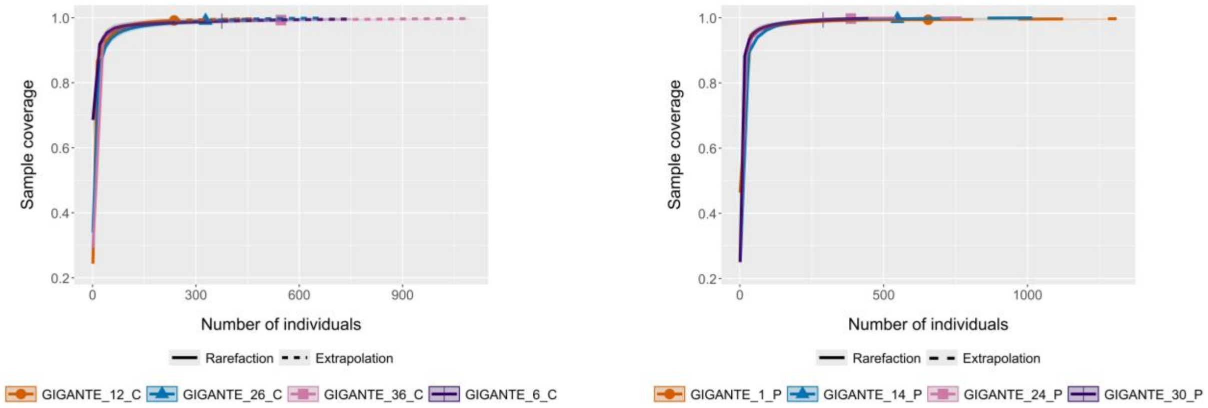


**Figure S13.** Sample completeness curves (with 95% confidence intervals as shaded areas) for fauna orders extracted from forest litter collected from the control and +P treatments at the Gigante peninsula forest fertilisation experiment in central Panama. The eight plots are presented across two separate panels for ease of visualisation; control plots are presented in the panel on the left, fertilised plots are presented in the panel on the right.

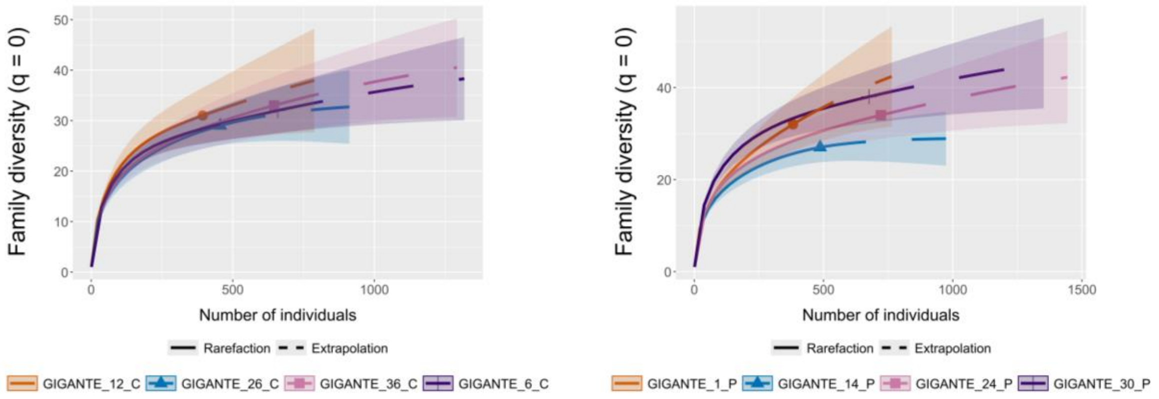


**Figure S14.** Sample size-based rarefaction and extrapolation sampling curves (with 95% confidence intervals as shaded areas) for Coleoptera family richness (q = 0) in ground-based flight-intercept traps installed in the control and +P treatments of the Gigante peninsula forest fertilisation experiment in central Panama. The eight plots are presented across two separate panels for ease of visualisation; control plots are presented in the panel on the left, fertilised plots are presented in the panel on the right.

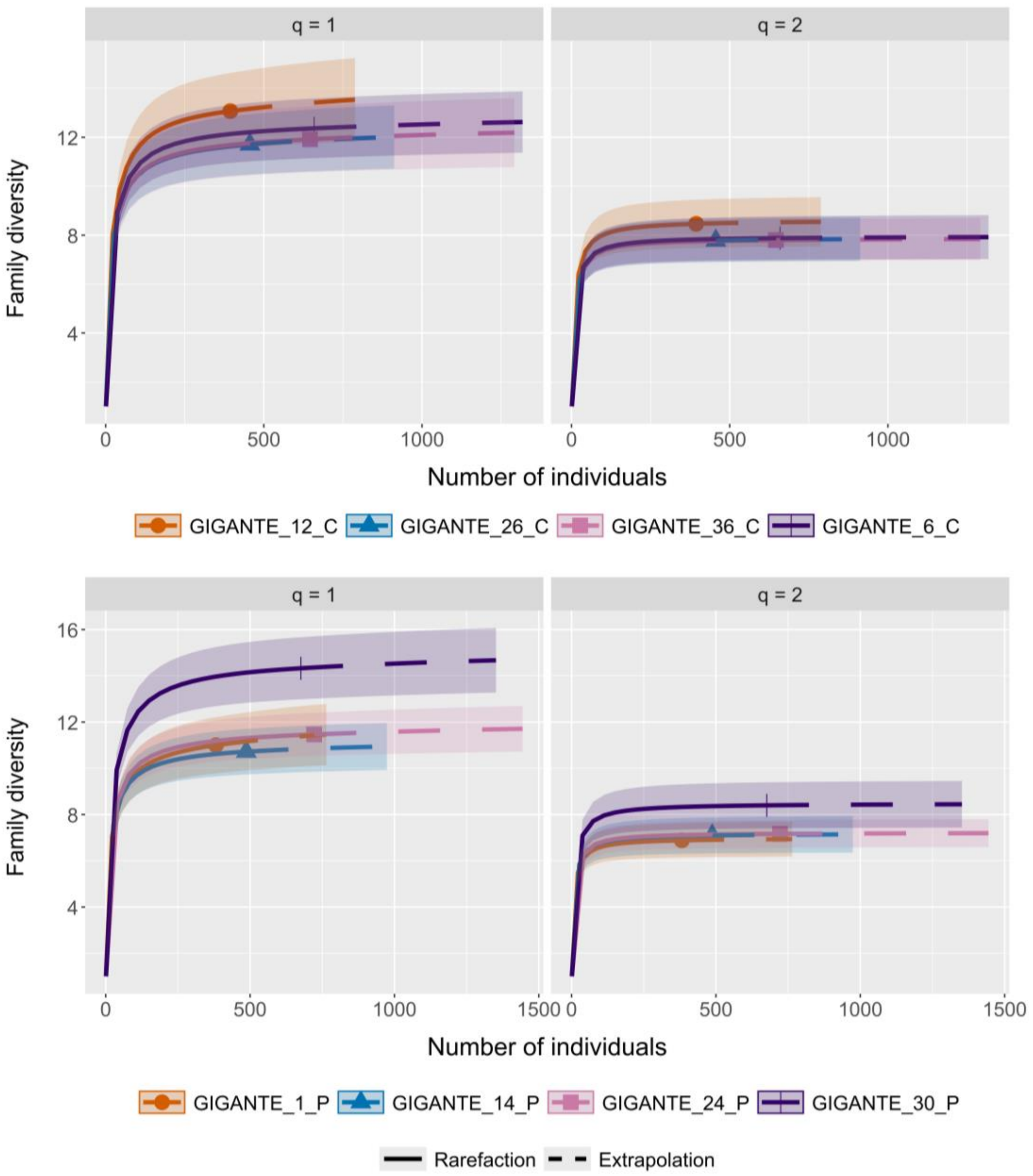


**Figure S15.** Sample size-based rarefaction and extrapolation sampling curves (with 95% confidence intervals as shaded areas) for Coleoptera family Shannon diversity (q = 1) and Simpson diversity (q = 2) in ground-based flight-intercept traps installed in the control and +P treatments of the Gigante peninsula forest fertilisation experiment in central Panama. The eight plots are presented across two separate panels (upper and lower panels) for ease of visualisation; control plots are presented in the panel on the left, fertilised plots are presented in the panel on the right.

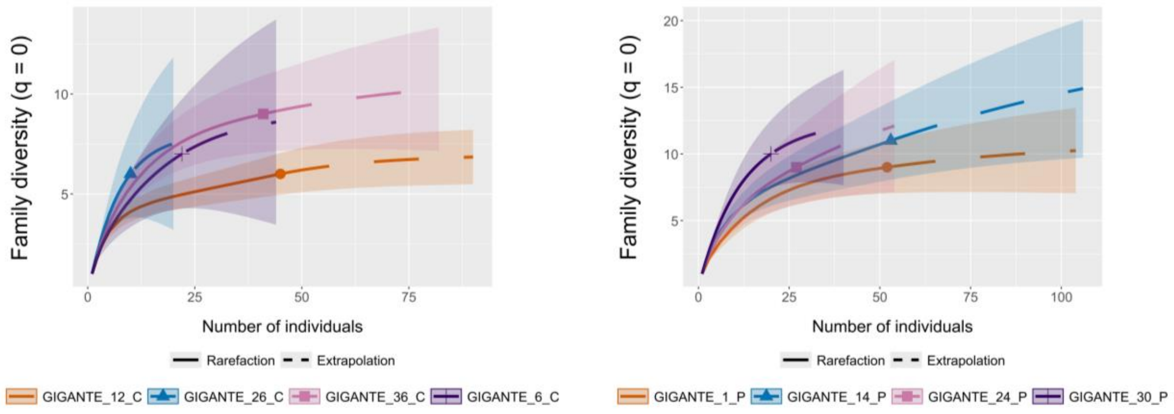


**Figure S16.** Sample size-based rarefaction and extrapolation sampling curves (with 95% confidence intervals as shaded areas) for Coleoptera family richness (q = 0) in extracts of forest litter collected from the control and +P treatments of the Gigante peninsula forest fertilisation experiment in central Panama. The eight plots are presented across two separate panels (upper and lower panels) for ease of visualisation; control plots are presented in the panel on the left, fertilised plots are presented in the panel on the right.

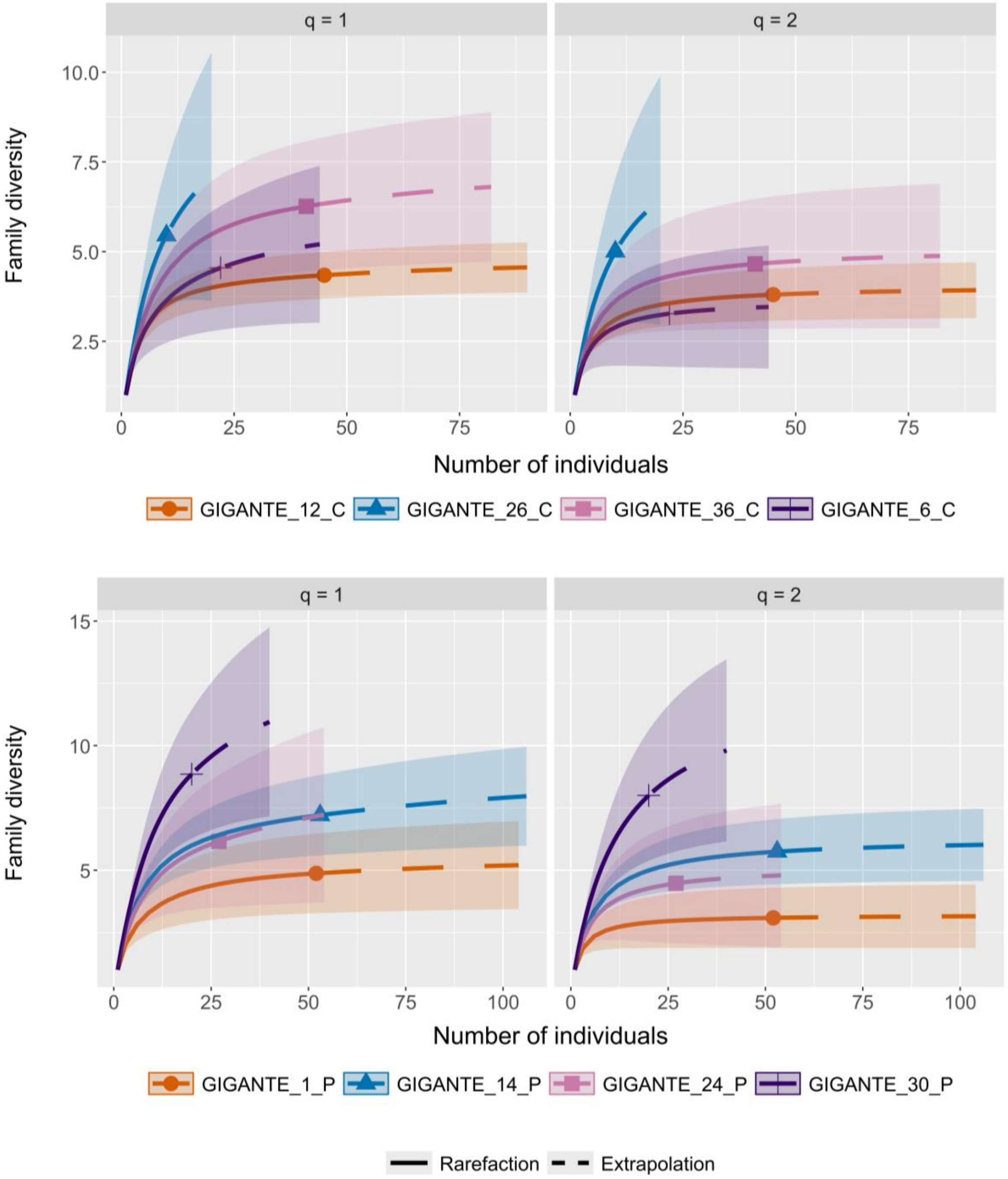


**Figure S17.** Sample size-based rarefaction and extrapolation sampling curves (with 95% confidence intervals as shaded areas) for Coleoptera family Shannon diversity (q = 1) and Simpson diversity (q = 2) in extracts of forest litter collected from the control and +P treatments of the Gigante peninsula forest fertilisation experiment in central Panama. The eight plots are presented across two separate panels (upper and lower panels) for ease of visualisation; control plots are presented in the panel on the left, fertilised plots are presented in the panel on the right.

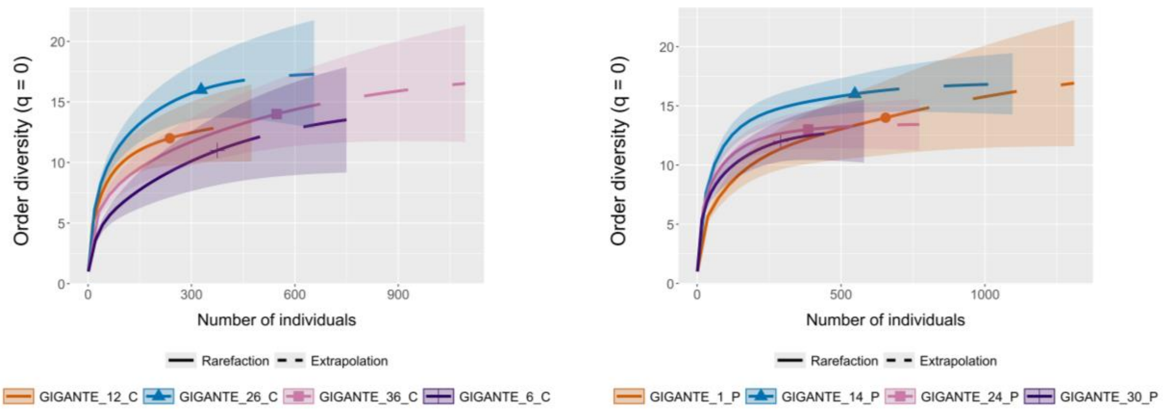


**Figure S18.** Sample size-based rarefaction and extrapolation sampling curves (with 95% confidence intervals as shaded areas) for order-level richness (q = 0) of fauna extracted from forest litter collected from the control and +P treatments of the Gigante peninsula forest fertilisation experiment in central Panama. The eight plots are presented across two separate panels (upper and lower panels) for ease of visualisation; control plots are presented in the panel on the left, fertilised plots are presented in the panel on the right.

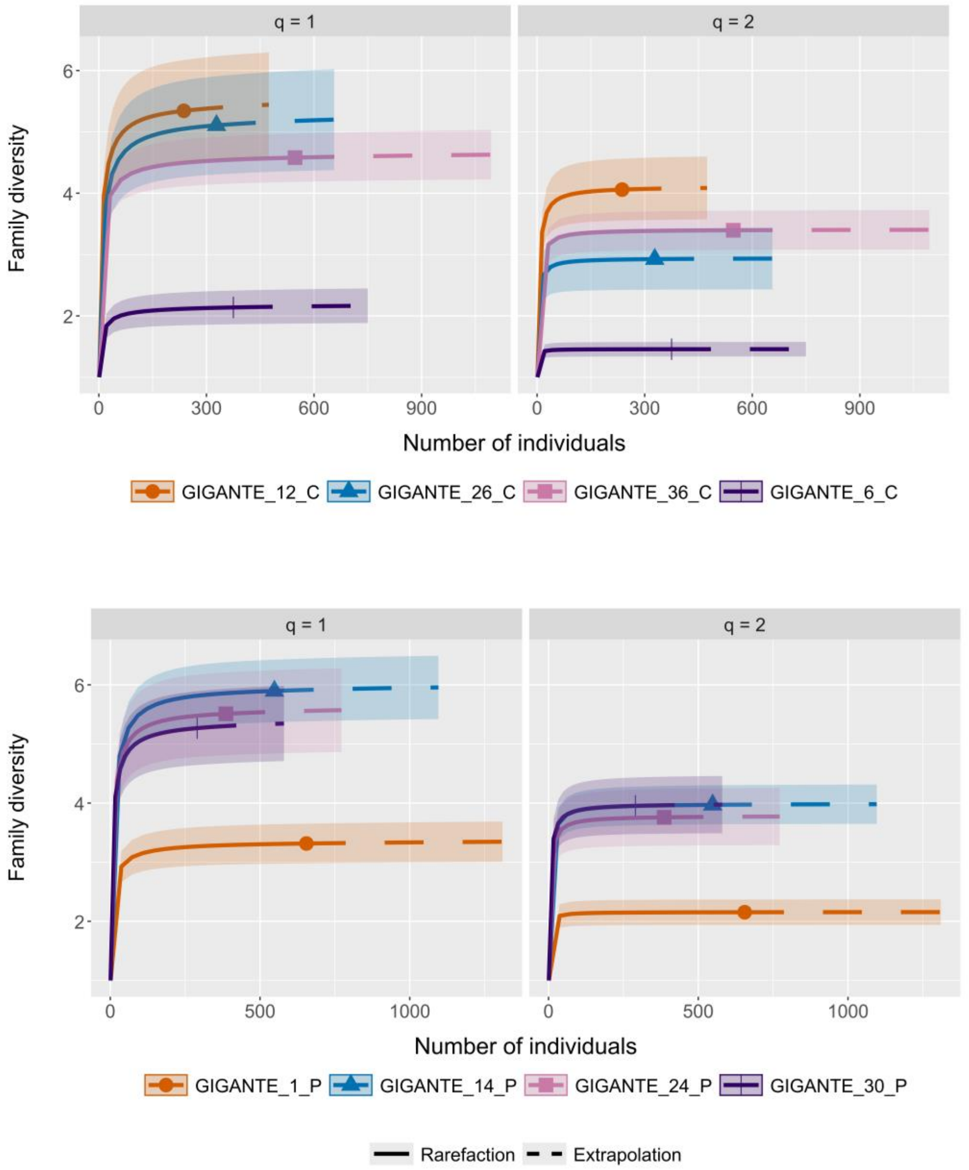


**Figure S19.** Sample size-based rarefaction and extrapolation sampling curves (with 95% confidence intervals as shaded areas) for order-level Shannon diversity (q = 1) and Simpson diversity (q = 2) of fauna extracted from forest litter collected from the control and +P treatments of the Gigante peninsula forest fertilisation experiment in central Panama. The eight plots are presented across two separate panels (upper and lower panels) for ease of visualisation; control plots are presented in the panel on the left, fertilised plots are presented in the panel on the right.

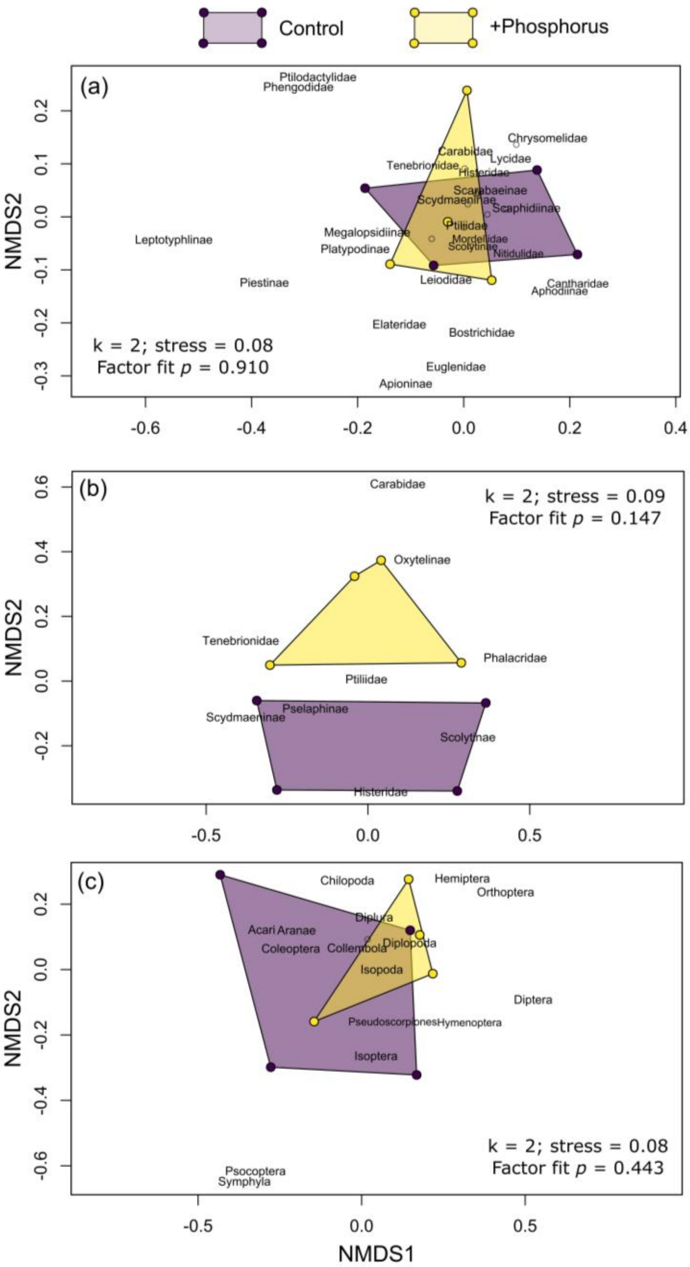


**Figure S20.** Non-metric multidimensional scaling [NMDS] and associated factor fitting analysis examining relationships between soil P fertilisation treatment and the assemblage composition of (a) Coleoptera families caught in flight-intercept traps, (b) Coleoptera families extracted from forest litter, and (c) arthropods orders extracted from forest litter in the lowland tropical forest of Gigante peninsula in central Panama.

**Table S1.** Summary of outputs of linear models used to evaluate the significance of soil phosphorus (P) indices as predictors of understorey arthropod abundance and diversity across a landscape-scale gradient of soil fertility in the lowland tropical forests of central Panama.

| *Response* | *Error distribution* | *P index* | *Intercept* | *Slope estimate* | *Slope z or t value* | *Pr(>\|z or t\|)* |
|---|---|---|---|---|---|---|
| FIT† coleoptera abundance | Negative binomial | Resin P | 7.76 | -0.1 | -0.9 | 0.388 |
| | | Mehlich P | 7.76 | -0.1 | -0.6 | 0.550 |
| | | Total P | 7.76 | 0.002 | 0.0 | 0.988 |
| FIT coleoptera Chao family richness | Gaussian | Resin P | 45.1 | 2.1 | 0.4 | 0.675 |
| | | Mehlich P | 45.1 | -0.9 | -0.2 | 0.852 |
| | | Total P | 45.1 | -1.9 | -0.4 | 0.708 |
| FIT coleoptera Chao family diversity (Shannon) | Gaussian | Resin P | 2.2 | -0.01 | -0.1 | 0.900 |
| | | Mehlich P | 2.2 | -0.04 | -0.4 | 0.706 |
| | | Total P | 2.2 | -0.03 | -0.3 | 0.762 |
| FIT coleoptera Chao family diversity (Simpson) | Gaussian | Resin P | 0.8 | 0.01 | 0.3 | 0.773 |
| | | Mehlich P | 0.8 | 0.002 | 0.1 | 0.939 |
| | | Total P | 0.8 | 0.01 | 0.2 | 0.814 |
| Tullgren coleoptera abundance | Negative binomial | Resin P | 4.4 | 0.4 | 2.5 | 0.012 * |
| | | Mehlich P | 4.4 | 0.3 | 1.9 | 0.061 . |
| | | Total P | 4.4 | 0.4 | 2.8 | 0.005 ** |
| Tullgren coleoptera Chao family richness | Gaussian | Resin P | 16.2 | -1.2 | -1.7 | 0.136 |
| | | Mehlich P | 16.2 | -1.4 | -2.0 | 0.087 . |
| | | Total P | 16.2 | -1.4 | -2.0 | 0.085 . |
| Tullgren coleoptera Chao family diversity (Shannon) | Gaussian | Resin P | 2.2 | 0.01 | 0.1 | 0.886 |
| | | Mehlich P | 2.2 | 0 | -0.1 | 0.904 |
| | | Total P | 2.2 | 0 | 0.2 | 0.814 |
| Tullgren coleoptera Chao family diversity (Simpson) | Gaussian | Resin P | 0.8 | 0 | 0.6 | 0.543 |
| | | Mehlich P | 0.8 | 0 | 0.4 | 0.730 |
| | | Total P | 0.8 | 0 | 0.7 | 0.487 |
| Tullgren fauna abundance | Negative binomial | Resin P | 7.1 | 0.2 | 2.2 | 0.030 * |
| | | Mehlich P | 7.1 | 0.1 | 1.3 | 0.197 |
| | | Total P | 7.1 | 0.3 | 2.9 | 0.004 ** |
| Tullgren fauna Chao richness ("order" level) | Gaussian | Resin P | 17 | 0.4 | 0.6 | 0.590 |
| | | Mehlich P | 17 | 0.6 | 0.7 | 0.490 |
| | | Total P | 17 | 0.3 | 0.4 | 0.674 |
| Tullgren fauna Chao diversity (Shannon; "order level") | Gaussian | Resin P | 1.5 | 0 | -0.1 | 0.952 |
| | | Mehlich P | 1.5 | 0 | 0.4 | 0.711 |
| | | Total P | 1.5 | 0.1 | 0.5 | 0.620 |
| Tullgren fauna Chao diversity (Simpson; "order" level) | Gaussian | Resin P | 0.6 | 0 | -0.2 | 0.877 |
| | | Mehlich P | 0.6 | 0 | 0.3 | 0.792 |
| | | Total P | 0.6 | 0 | 0.4 | 0.712 |

†FIT = flight-intercept trap.

**Table S2.** Summary of outputs of multivariate abundance models used to evaluate the significance of soil phosphorus (P) indices as predictors of the taxon-specific abundances understorey arthropods across a landscape-scale gradient of soil fertility in the lowland tropical forests of central Panama.

| *Response* | *P index* | Wald statistic | Pr(>Wald) |
|---|---|---|---|
| FIT† coleoptera families | Resin P | 9.8 | 0.088 . |
| | Mehlich P | 11.6 | 0.039 * |
| | Total P | 10.5 | 0.043 * |
| Tullgren coleoptera families | Resin P | 7.1 | 0.034 * |
| | Mehlich P | 6.2 | 0.114 |
| | Total P | 7.4 | 0.018 * |
| Tullgren fauna "orders" | Resin P | 8.1 | 0.019 * |
| | Mehlich P | 8.7 | 0.021 * |
| | Total P | 10.4 | 0.003 ** |
| FIT Coleoptera trophic guilds | Resin P | 2.6 | 0.523 |
| | Mehlich P | 4 | 0.16 |
| | Total P | 3.4 | 0.284 |
| Tullgren Coleoptera trophic guilds | Resin P | 4.1 | 0.091 . |
| | Mehlich P | 3.6 | 0.171 |
| | Total P | 5 | 0.031 * |
| FIT random 36 families | Total P | 9.7 | 0.036 * |
| Tullgren random 9 families | Total P | 5.5 | 0.026 * |
| Tullgren random 17 orders | Total P | 9.5 | 0.007 ** |

†FIT = flight-intercept trap.

**Table S3**. Summary of outputs of 'ordisurf' surface fitting analysis used to evaluate the associations between the composition of understorey arthropod assemblages and soil phosphorus (P) indices across a landscape-scale gradient of soil fertility in the lowland tropical forests of central Panama.

| *Response* | *P index* | *F*-statistic | Approximate significance of smooth term (*p*) |
|---|---|---|---|
| FIT† coleoptera families | Resin P | 2.3 | 0.044 * |
| | Mehlich P | 1.4 | 0.062 . |
| | Total P | 3.2 | 0.027 * |
| | Total P (unscaled, no log-transform) | 3.5 | 0.041 |
| Tullgren coleoptera families | Resin P | 0.3 | 0.267 |
| | Mehlich P | 0.9 | 0.133 |
| | Total P | 1.2 | 0.088 . |
| | Total P (unscaled, no log-transform) | 2.7 | 0.068 . |
| Tullgren fauna "orders" | Resin P | 5891205 | |
| | Mehlich P | 184479 | |
| | Total P | 786442 | |
| | Total P (unscaled, no log-transform) | 1169369 | |

†FIT = flight-intercept trap. Note that, due to extreme *F*-statistic values for analyses of litter-extracted fauna orders, we regard the associated *p* values as unreliable and have thus excluded them from this result summary.

**Table S4.** Summary of results of one-way ANOVAs testing the effects of long-term phosphorus fertilisation on the abundance and diversity of understorey arthropods in the lowland tropical forests of Gigante Peninsula, central Panama.

| *Response* | *Error distribution* | *Intercept* | *Slope estimate* | *Slope z or t value* | *Pr(>\|z or t\|)* |
|---|---|---|---|---|---|
| FIT[†] coleoptera abundance | Negative binomial | 6.3 | 0.1 | 0.3 | 0.761 |
| FIT coleoptera Chao family richness | Gaussian | 47.6 | 4 | 0.4 | 0.678 |
| FIT coleoptera Chao family diversity (Shannon) | Gaussian | 2.6 | 0 | -0.5 | 0.633 |
| FIT coleoptera Chao family diversity (Simpson) | Gaussian | 0.9 | 0 | -1.7 | 0.142 |
| Tullgren coleoptera abundance | Negative binomial | 3.3 | 0.2 | 0.6 | 0.559 |
| Tullgren coleoptera Chao family richness | Gaussian | 8.8 | 6.5 | 2.7 | 0.034 * |
| Tullgren coleoptera Chao family diversity (Shannon) | Gaussian | 1.8 | 0.3 | 1.3 | 0.231 |
| Tullgren coleoptera Chao family diversity (Simpson) | Gaussian | 0.8 | 0 | 0.3 | 0.741 |
| Tullgren fauna abundance | Negative binomial | 5.9 | 0.2 | 1.1 | 0.284 |
| Tullgren fauna Chao richness ("order" level) | Gaussian | 16.1 | -0.2 | -0.1 | 0.901 |
| Tullgren fauna Chao diversity (Shannon; "order level") | Gaussian | 1.4 | 0.2 | 0.7 | 0.498 |
| Tullgren fauna Chao diversity (Simpson; "order" level) | Gaussian | 0.6 | 0.1 | 0.7 | 0.488 |

[†]FIT = flight-intercept trap.

**Table S5.** Summary of results of multivariate abundance analyses testing the effects of long-term phosphorus fertilisation on the taxon-specific abundances of understorey arthropods in the lowland tropical forest of Gigante Peninsula, central Panama.

| *Response* | Wald statistic | Pr(>Wald) |
|---|---|---|
| FIT[†] coleoptera families | 8.5 | 0.128 |
| Tullgren coleoptera families | 4.3 | 0.177 |
| Tullgren fauna "orders" | 4.1 | 0.413 |
| FIT Coleoptera trophic guilds | 3.8 | 0.264 |
| Tullgren Coleoptera trophic guilds | 3.3 | 0.285 |

[†]FIT = flight-intercept trap.
